# OrthoFinder: improved phylogenetic orthology inference with enhanced accuracy and scalability

**DOI:** 10.1038/s41592-026-03126-6

**Published:** 2026-06-09

**Authors:** David M. Emms, Yi Liu, Laurence Belcher, Jonathan Holmes, Steven Kelly

**Affiliations:** Department of Biology, University of Oxford, Oxford, UK

**Keywords:** Software, Genome informatics, Evolution, Evolutionary biology, Comparative genomics

## Abstract

Here we present a major advancement of the OrthoFinder method. This extends OrthoFinder’s high-accuracy comparative genomic framework to provide substantially enhanced scalability and accuracy. Specifically, we show that enhanced phylogenetic delineation of orthogroups provides a 7% relative increase in orthogroup inference accuracy. We further demonstrate that a new gene assignment method substantially reduces overall runtime RAM usage without compromising accuracy. The latest version of OrthoFinder is available via GitHub at https://github.com/OrthoFinder/OrthoFinder.

## Abstract (web summary)

The updated OrthoFinder v3 software boosts accuracy and scalability in phylogenetic orthology inference with massive and diverse datasets.

## Main

Inferring orthology between biological sequences is fundamental to contemporary biological research. It provides the foundation for studying the evolution and diversity of life on Earth, as well as the framework for transferring biological knowledge between species. Given the central importance of orthology inference to biological research, it has been the subject of extensive methodological development for more than 40 years^1–4^. Diverse approaches to the computational challenge of identifying related genes in different species has resulted in a broad array of methods with varied performance characteristics when applied to diverse datasets^3,5,6^.

Although orthology inference has been subject to substantial improvements since the advent of the first automated methods, one of the major challenges facing method development in this field is how to achieve high orthology inference accuracy at scale^7,8^. This challenge has become more pressing in recent years with genome sequencing projects such as The Darwin Tree of Life^9^ and The Earth BioGenome Project^10^, which collectively aim to sequence, assemble and annotate the reference genomes for all two million known species of eukaryote. These pioneering efforts, combined with widespread sequencing efforts that are distributed across many biological communities, are creating an unprecedented scalability challenge for automated orthology inference methods^4,11^. Thousands of genomes are already available, and millions more are likely to become available; there is a clear need to accurately and efficiently analyze these resources to maximize their value.

There are several distinct computation bottlenecks for inferring orthology for large numbers of species. For example, the entry point to the majority of inference methods is an all-versus-all sequence similarity search from which orthology relationships can be inferred^12^. Substantial efforts have been focused at increasing the computational efficiency of individual sequence similarity searches, with methods such as DIAMOND^13^, Usearch^14^ and mmseqs^15^ greatly advancing the capability to efficiently identify similar sequences in large databases. Despite major improvements, all-versus-all sequence searches have an order of *n*^2^ time complexity (where *n* is the number of species under consideration) and are therefore not well suited to orthology inference as the number of species increases. Thus, alternative orthology inference approaches that retain high accuracy while improving scalability are required to realize the full value of global sequencing efforts.

Previously we developed the OrthoFinder method for phylogenetic orthology inference^12,16^. OrthoFinder first identifies orthogroups, the sets of genes that each descend from a single gene in the last common ancestor of a group of species. OrthoFinder then infers a gene tree for each orthogroup and analyzes these gene trees to identify the rooted species tree^17,18^. OrthoFinder also identifies all gene duplication events in the complete set of gene trees and analyzes this information in the context of the species tree, providing gene tree- and species tree-level analyses of gene duplication events. Finally, OrthoFinder analyzes this cohort of phylogenetic information to identify the complete set of orthologs between all species and provide a suite of comparative genomics statistics. The phylogenetic approach developed in OrthoFinder provided a step change for comparative genomics, enabling the transition from similarity score-based approximations of orthology to tree-based phylogenetic relationships between genes. In recent work, we showed that it was possible to rapidly and accurately place individual sequences into a database of phylogenetic trees and biological sequences produced by an OrthoFinder search^19^. This approach provided a rapid phylogenetic framework through which individual genes could be added to an existing orthology inference run without incurring a computationally costly all-versus-all reanalysis of existing species. We hypothesized that it should be possible to extend this single-gene approach to support the addition of sets of species and thereby achieve improved scalability without compromising accuracy.

Here, we present a major update to OrthoFinder that substantially enhances the scalability of the method. We show that it is possible to rapidly search and assign sequence sets from large numbers of species to a phylogenetically partitioned and structured database of biological sequences in near linear time. We demonstrate that using such an accelerated search functionality does not compromise orthology inference accuracy. Moreover, through advancements in phylogenetic interrogation of orthogroups, we show that it is possible to achieve scalable inference with higher accuracy than any competitor method or previous versions of OrthoFinder. Finally, we show that the latest version of OrthoFinder is scalable to thousands of genomes on conventional computing resources. As before, the updated version of OrthoFinder is accurate and customizable, and runs with a simple command using only protein sequences as input.

## Results

### Improved phylogenetic delineation of orthogroups

**Fig. 1:**
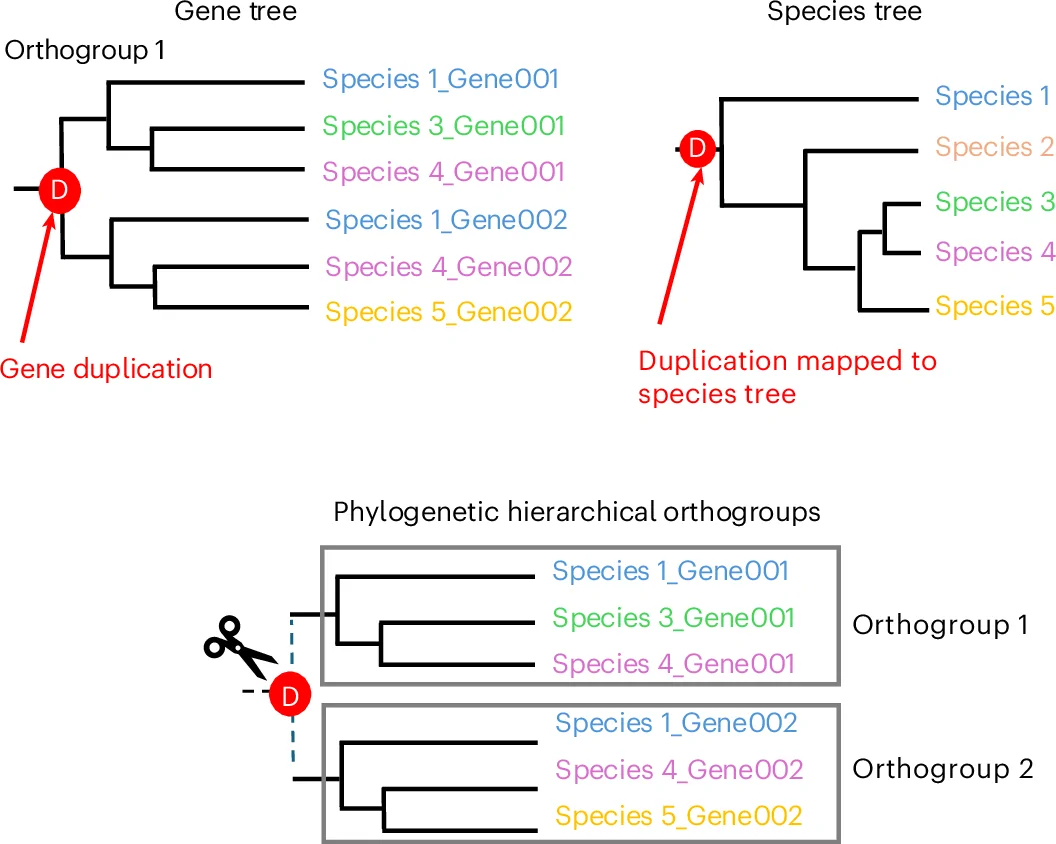
The improved phylogenetic delineation of orthogroups. OrthoFinder reconciles gene trees with the species tree to identify duplication events within putative MCL orthogroups. When a duplication event is identified to have occurred before the divergence of the species set under consideration, then the orthogroup is split into two (or more) independent orthogroups, ensuring that each resulting orthogroup contains only genes descended from a single gene in the last common ancestor of all species (the definition of an orthogroup).

In previous versions of OrthoFinder, orthologs were defined phylogenetically, whereas orthogroups were defined solely based on Markov Cluster Algorithm (MCL) clustering of sequence similarity search results^12,16^. While this method does correct for interspecies divergence, it does not leverage the phylogenetic relationship between the species represented in each orthogroup, and thus does not always identify the true extent of an orthogroup. Errors in orthogroup delineation at this stage propagate through all subsequent steps of the analysis, causing collateral errors in orthology inference. To address this, we developed and implemented a phylogenetic re-evaluation of orthogroup membership (Fig. 1). First, MCL clustering is performed as in OrthoFinder v2, and a gene tree is inferred for each orthogroup. Next, OrthoFinder applies its high-accuracy gene tree species tree reconciliation algorithm^12^ to identify and map all gene duplication events in each gene tree. By definition, an orthogroup should not contain genes arising from ‘ancient’ duplications that predate the root of the species tree; any such duplications indicate that the genes have descended from multiple independent genes present in the last common ancestor of the set of species under consideration. OrthoFinder splits all gene trees at such ancient gene duplication nodes and, in doing so, creates a new set of revised phylogenetically defined orthogroups, which are then recorded and used for all subsequent downstream analyses. These orthogroups correspond to the root node of the species tree; however OrthoFinder also conducts the phylogenetic delineation analysis at every ancestral node in the species tree. This creates orthogroups for each node in the species tree. This new step splits erroneously fused orthogroups, and prunes sequences that do not adhere to the phylogenetic definition of the orthogroup (Fig. 1).

To test the impact of this approach on orthogroup inference accuracy, we compared this new version of OrthoFinder with a set of alternative methods on the OrthoBench dataset of expert-curated reference orthogroups^20,21^. We deployed OrthoFinder in its two most commonly used implementations. One implementation uses multiple sequence alignment and maximum likelihood tree inference to infer orthogroup trees and is provided with a precomputed species tree (OF3_Align_ST), and the other implementation uses the DendroBLAST method to bypass the requirement to construct alignments and alignment-based trees^22^ (OF3_DB) which is the default implementation in OrthoFinder v2. We also included the new scalable version of OrthoFinder v3 (OF3_Linear) which is discussed in detail in the following section. The list of comparator methods included Broccoli^23^, OrthoHMM^24^, SonicParanoid2^7^, OrthoMCL^25^, ProteinOrtho^26^, Hieranoid^27^ and FastOMA^8^.

**Fig. 2:**
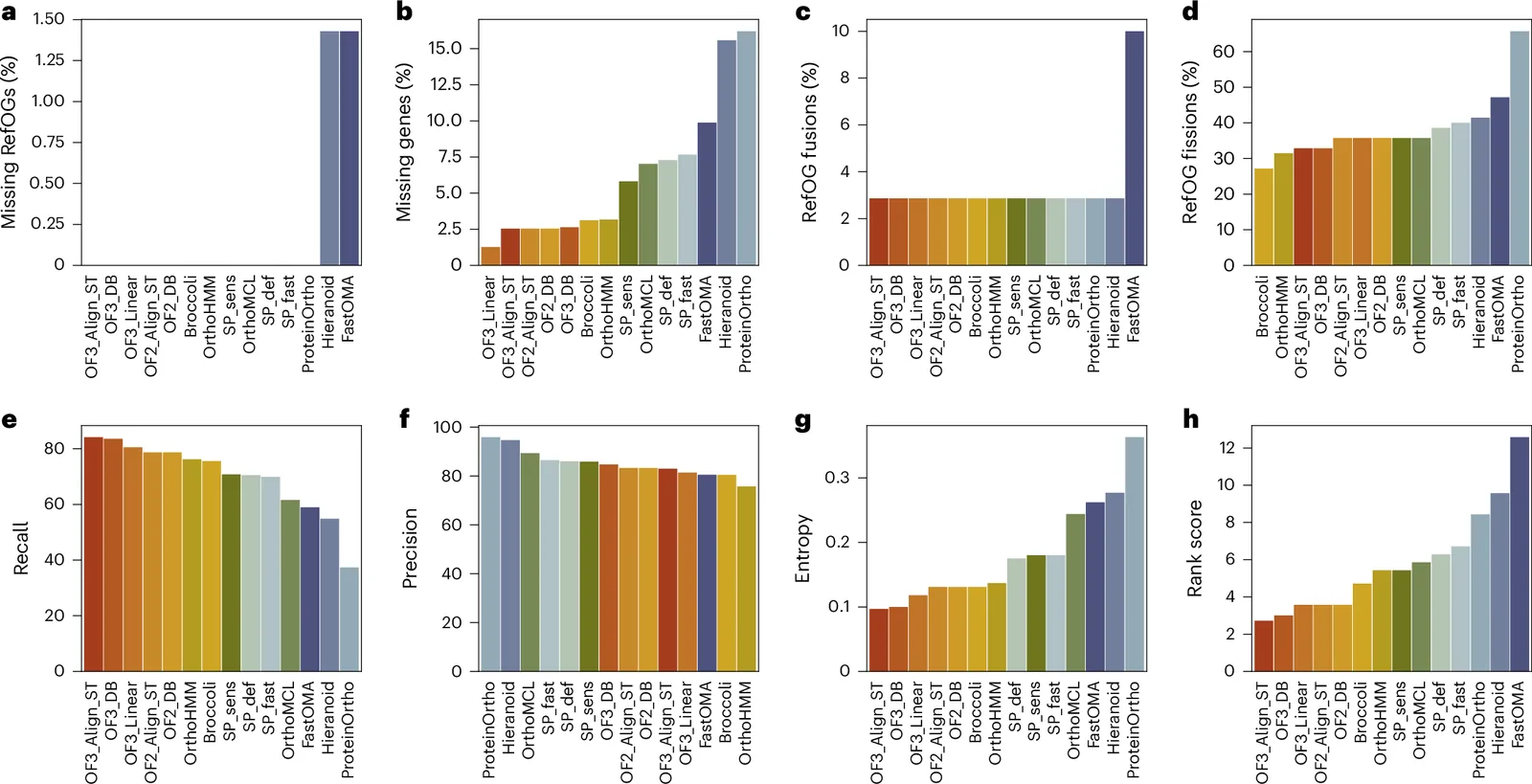
Comparative analysis of different orthogroup inference tools on the OrthoBench dataset. **a**–**g**, Performance across seven orthology prediction metrics: percentage of missing reference orthogroups (**a**); percentage of missing genes (**b**); fusion error, measured as the percentage of reference orthogroups fused with one or more other reference orthogroups in the same predicted orthogroup (**c**); fission error, measured as the percentage of reference orthogroups split across two or more predicted orthogroups (**d**); recall (**e**); precision (**f**) and entropy, which measures the dispersion of genes from each reference orthogroup across predicted orthogroups (**g**). **h**, Overall rank score across the seven metrics shown in **a**–**g**, calculated by ranking each method for each metric and averaging these ranks. Lower values indicate better performance for error-based metrics in **a**–**d** and **g**, whereas higher values indicate better performance for recall and precision in **e** and **f**. For the rank score in **h**, lower values indicate better overall performance.

For each method, we calculated seven measures of accuracy: (1) the percentage of missing reference orthogroups, (2) the percentage of missing genes, (3) the percentage of incorrect orthogroup fusion events, (4) the percentage of incorrect orthogroup fission events, and (5) recall, (6) precision and (7) entropy. Entropy is the measure of reference orthogroup fragmentation, with higher values indicating greater fragmentation. We then calculated an overall rank-based score by evaluating the relative performance of each method across all seven measures. This revealed that OrthoFinder v3 (in either implementation) outperformed all other orthogroup inference methods including OrthoFinder v2 (Fig. 2). OrthoFinder v3 had the highest recall, lowest fraction of missing genes and lowest entropy. Moreover, OrthoFinder v3 had 5–7% higher accuracy (*f*-score harmonic mean of recall and precision; Supplementary Table 1.1) than the previous version of OrthoFinder run with identical settings (OF2_Align_ST and OF2_DB; Fig. 2). While OrthoFinder v3 achieved lower precision than several other methods, it showed substantially higher recall and consequently had a much lower rate of missing data. For example, while SonicParanoid2 (SP_def) showed a 3.7% higher precision than OrthoFinder v3 (OF3_Align_ST), the recall of OrthoFinder v3 was 19% higher. Almost all tools were identical in the production of fused reference orthogroups (RefOG), with the exception of FastOMA, which erroneously fused 3.5 times more reference orthogroups than other methods (10% compared with 2.85%). The spread of reference orthogroup fissions events across the tools varied, with low-precision tools such as Broccoli and OrthoHMM making relatively few RefOG fissions. Analysis of the entropy scores, which are similar to a weighted combination of fission and fusion events, revealed that OrthoFinder tends to produce orthogroups whose sizes and membership most accurately recapture the reference dataset (Fig. 2). Therefore, the introduction of the phylogenetic delimitation of orthogroups improves overall orthogroup inference accuracy, and OrthoFinder v3 is the most accurate method currently available.

### Achieving enhanced scalability without compromising accuracy

**Fig. 3:**
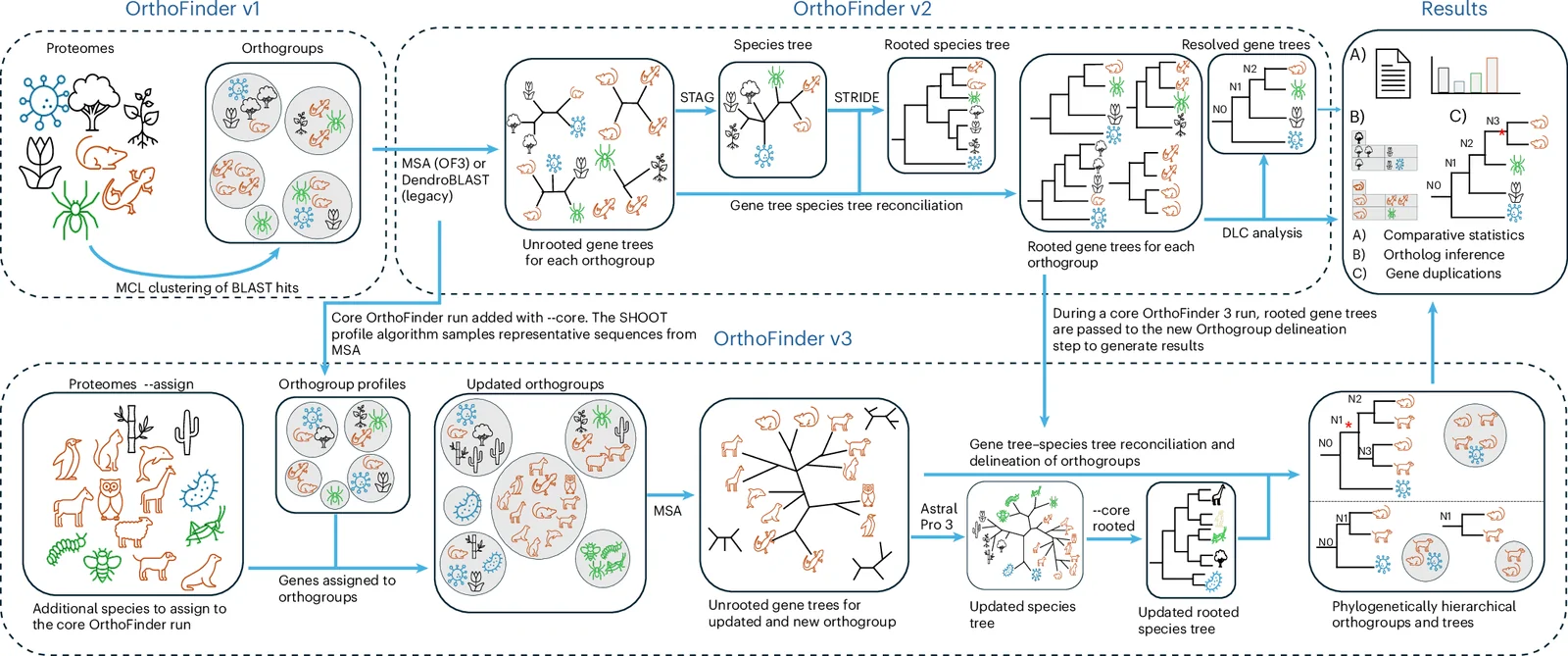
The OrthoFinder v3 workflow. The first step of OrthoFinder v3 is to perform a conventional OrthoFinder run on a set of core proteomes. The second step then uses an adapted version of the SHOOT profiling algorithm to assign genes from additional species to these core orthogroups and identify new orthogroups not present in the initial input gene set. Gene trees are then inferred and used to infer a revised species tree. A Duplication-Loss-Coalescence (DLC) analysis is then performed on the rooted gene trees to identify orthologs and gene duplication events (which are mapped to their location on both the gene and species tree). All results files are then revised and updated to reflect the phylogenetic hierarchical orthogroups identified from this.

In addition to improvements in the phylogenetic definition of orthogroups, we also developed a novel implementation of OrthoFinder to improve the scalability of the method. This is achieved through adaptation and further development of the SHOOT profile algorithm^19^, which enables rapid addition of new species to existing OrthoFinder runs. This novel scalable version of OrthoFinder is implemented as a two-step process (Fig. 3). Before starting, the input species set are partitioned into two non-overlapping subsets: a ‘core’ subset and an ‘assign’ subset. It is recommended that the core subset contain fewer than 100 species for analysis on conventional computing resources. The assign subset can be substantially larger, as described below. The first step of this new implementation requires that a conventional OrthoFinder run is performed on the core subset. This creates a phylogenetically partitioned and structured reference database of biological sequences^19^ that is then used as input for the second stage of the algorithm. The species from the assign subset are then rapidly assigned to the correct orthogroup in the reference database, irrespective of the number of sequences contained within each core orthogroup. Following completion of the assignment step, OrthoFinder’s rapid phylogeny-based orthogroup and ortholog inference steps are performed to produce an expanded rooted species tree, gene trees, phylogenetically determined orthogroups, orthologs, gene duplication events and comparative genomic statistics (Fig. 3).

**Fig. 4:**
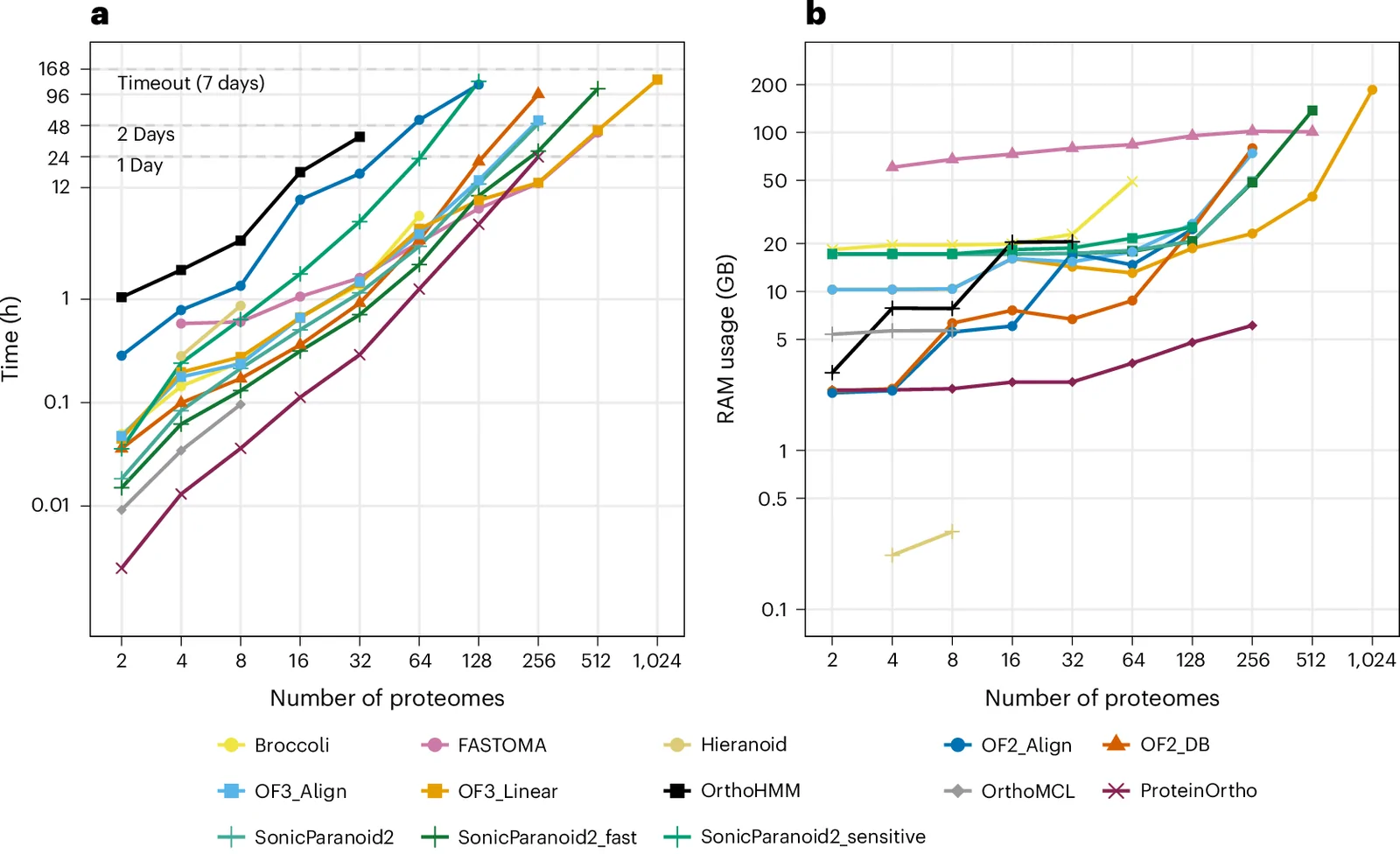
A comparison of runtime and peak memory usage for orthogroup inference tools. **a**,**b**, Time (log scale) (**a**) and peak RAM usage (**b**) required to run different orthology prediction tools across increasing numbers of input proteomes (from 2 to 1,024). When the number of proteomes is ≤64, OrthoFinder v3 was run using the OrthoFinder v2 workflow. When the number of proteomes is >64, OrthoFinder v3 is used to ‘assign’ the remaining proteome to the core set of 64 proteomes. Runtime for OrthoFinder v3 with >64 proteomes is the sum of runtime of the ‘core’ run and runtime of the ‘assign’ run. An additional plot showing variation in runtime for the same dataset for both OrthoFinder v3 Linear and FastOMA is shown in Supplementary Fig. 2.

To test the impact on scalability of this new workflow, OrthoFinder v3 was compared against the set of commonly used orthology inference tools. The time each method took to complete analyses of datasets of different sizes was measured, unless a timeout of 7 days was reached before completion. Peak memory usage was also calculated for each analysis. Each tool was run on a Linux server using advanced micro devices (AMD) processers, all tools were allocated 32 threads (16 cores) and up to 200 GB of random access memory (RAM) for each run. To test performance on a representative set of species, the Ensembl^28^ rapid release genomes (accessed 29 August 2024) were downloaded. Test datasets ranging in size from 2 to 1,024 species were compiled by sampling species from the Ensembl dataset using the phylogenetic diversity analyzer (PDA^29^; see the Methods for more details). All methods were run on the same datasets in their default implementations unless indicated otherwise in the methods or figure legends. OrthoFinder v3 was run using the standard OrthoFinder 2 workflow up to 64 species. Beyond 64 species, OrthoFinder v3 was run using the new scalable implementation, in which the first 64 species were taken as the core set and all other species were assigned accordingly. The final runtime for OrthoFinder v3 was calculated using the combined runtime for the core 64 species and the assigned dataset. OrthoFinder v3 linear addition outperformed all other tested methods, being the only method able to perform orthology inference on 1,024 species within the 7-day cutoff (Fig. 4a). OrthoFinder v3 completed this run in 128 h. SonicParanoid2 (fast mode) and FastOMA were the only two methods able to run 512 proteomes within the cutoff time. OrthoFinder v3 followed a near linear runtime trend for 128–1,024 species. This new implementation of OrthoFinder was faster than all other competitor methods tested and was substantially faster (8×) than OrthoFinder v2 on datasets containing more than 64 species, representing an important improvement in scalability over the previous version.

OrthoFinder v3 also performed well on memory usage (Fig. 4b). OrthoFinder v3 outperformed OrthoFinder v2 on datasets containing more than 128 species, with memory savings increasing as the number of species increased. For example, v3 linear shows a 3.4-fold decrease in RAM consumption for 256 species compared with v2 DendroBlast. On the largest datasets that other methods could complete, OrthoFinder v3 used approximately 4-fold lower peak memory consumption (Fig. 4b). OrthoFinder, along with many other orthology inference tools, utilizes parallel processing to accelerate parts of its workflow. We examined how varying thread count influenced runtime and memory usage for OF3_Linear and compared this with FastOMA. Both tools were executed on a 64-proteome dataset across a series of thread settings (4, 8, 16, 32, 64 and 128). For OF3_Linear, a core 16 proteomes were initially run, after which the remaining 48 proteomes were assigned. Full results are provided in Supplementary Fig. 1. Both tools exhibited comparable trends in runtime reduction and memory scaling as thread counts increased. Runtime decreased as threads increased up to 32, beyond which performance gains plateaued.

To demonstrate that the alternative implementation did not compromise orthogroup inference accuracy, the linear species addition method (OF3_Linear) was also tested on the OrthoBench data (Fig. 2). OrthoFinder v3 Linear only slightly underperformed compared with the nonscalable version, with slight decreases in some measures observed. However, this scalable version was more accurate than any implementation of OrthoFinder v2 and any other competitor methods (Fig. 2), supporting the use of this accelerated implementation of OrthoFinder in comparative genomic analyses.

To further test the scalability of OrthoFinder v3 for larger numbers of species, datasets containing 2,048 and 4,096 bacterial species were downloaded from Ensembl. Similarly to the eukaryotic dataset, a core of 64 species was first created from a species tree using PDA with the remaining species assigned to the core. FastOMA, the only other tool that was able to achieve a run of 512 proteome in under 7 days, was included as a comparator. OrthoFinder successfully completed the 2,048-species dataset in 50 h and the 4,096-species proteome dataset in 13 days and 15 h, with a memory consumption of 504 GB. FastOMA completed the 2,048-species bacterial dataset in 14 days but was unable to complete the 4,096-species dataset.

### OrthoFinder v3 performs accurate ortholog inference

We also tested the accuracy of the orthologs predicted by OrthoFinder v3 in multiple implementations using the Quest for Orthologs (QfO) benchmarking service^2–4^. QfO is the most widely used orthology benchmarking service, which evaluates a tool’s ability to accurately predict orthologs across a diverse set of taxa, including Archaea, Bacteria and eukaryotes. QfO gives many different measures of accuracy, including comparisons with expert-curated reference orthologs and the accuracy of species trees inferred from a set of orthologs. We used the most recent published set of reference proteomes, which consists of 78 species (23 bacteria, 48 eukaryotes and 7 archaea)^30^. We compared OrthoFinder v3 against the results obtained from 14 orthology inference tools.

**Fig. 5:**
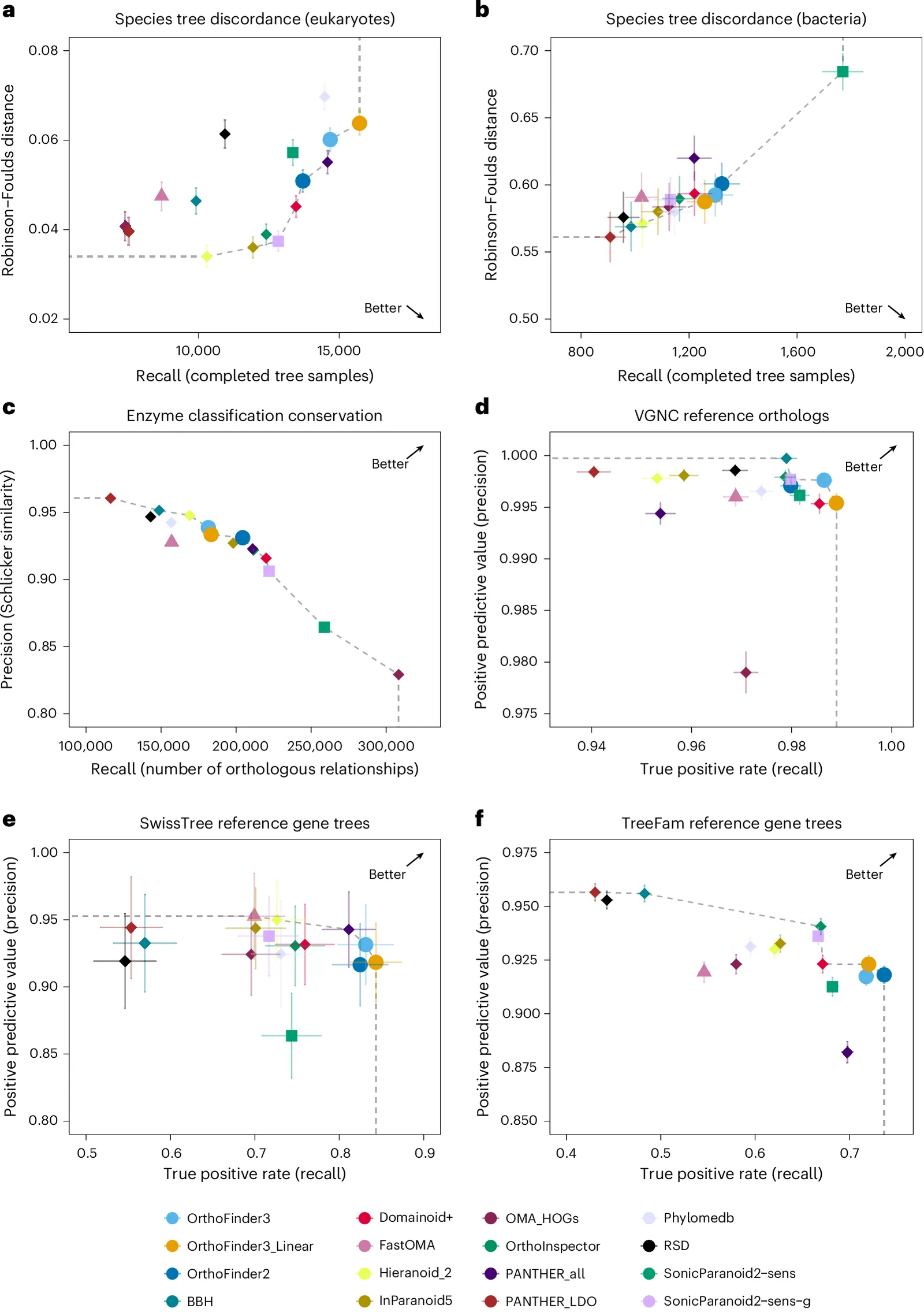
Comparative analysis of ortholog inference accuracy on the QfO dataset. Each panel shows a different benchmark from the QfO benchmarking service (2022 release). OrthoFinder is compared against other ortholog inference methods with publicly available results. **a**,**b**, Species tree discordance (Robinson–Foulds distance) versus recall across completed gene trees for eukaryotes (**a**) and bacteria (**b**). Points show mean values; vertical error bars indicate ± s.e.m. of Robinson–Foulds distance across completed tree samplings, and horizontal error bars indicate the approximate 95% interval for the number of completed tree samplings (*n* equals the number of completed tree samplings shown on the *x* axis). **c**, Conservation of enzyme classification across orthologs (Schlicker similarity) versus number of orthologous relationships. **d**, VGNC reference orthologs: precision versus recall. Points show proportions; error bars indicate approximate 95% binomial confidence intervals. The unit of analysis is an individual ortholog pair (*n* = 19,537 curated VGNC ortholog pairs for recall; precision is computed over predicted ortholog pairs). **e**,**f**, SwissTree (**e**) and TreeFam (**f**) reference gene trees: precision versus recall. Points show proportions; error bars indicate approximate 95% binomial confidence intervals. The unit of analysis is an individual ortholog pair (*n* = 10,416 and *n* = 79,818 ortholog pairs derived from SwissTree and TreeFam reference gene trees, respectively). Better-performing methods are toward the top right for precision measures (**c**–**f**) and bottom right for discordance measures (**a** and **b**).

Analysis of the resulting scores revealed that the new OrthoFinder v3 workflow for high scalability does not compromise accuracy (Fig. 5). OrthoFinder v3 is on the Pareto frontier for species tree discordance tests in both eukaryotes (Fig. 5a), and bacteria (Fig. 5b), demonstrating its ability to perform accurate orthology inference across different domains of life. The only other tool with scalability comparable to OrthoFinder v3 is FastOMA, but this method has substantially lower ortholog inference accuracy (Fig. 5). For example, while the Robinson–Foulds distance (measuring disagreement between true and inferred species trees) for eukaryotes is marginally worse in OrthoFinder v3 compared with FastOMA (0.06 versus 0.05), OrthoFinder v3 has an 80% higher recall (15,721 versus 8,686). Similarly, OrthoFinder v3 performed marginally better than FastOMA on the bacterial species tree discordance test in terms of Robinson–Foulds distance (0.590 versus 0.587), while again achieving a 23% higher recall. OrthoFinder v3 is on the Pareto frontier for the enzyme classification test (Fig. 5c), which measures how well predicted orthologs match to Enzyme Commission numbers (which are given depending on the chemical reaction they catalyze). OrthoFinder v3 is only outperformed in precision by tools that rely on precomputed databases (for example, OrthoInspector and PANTHER), and is better than FastOMA in both precision (0.933 versus 0.928) and recall (183,368 versus 157,049).

OrthoFinder v3 is on the Pareto frontier for all three of the human-curated reference sets (Fig. 5d–f), scoring particularly well on recall. OrthoFinder v3 achieved the highest recall of all methods on both the Vertebrate Gene Nomenclature Committee (VGNC) and SwissTree human-curated reference sets and was only marginally outperformed in recall on the TreeFam dataset by OrthoFinder v2 (0.72 versus 0.74). In summary, similar to the high accuracy of its orthogroup inference, OrthoFinder v3 also exhibits high accuracy ortholog inference across multiple benchmark tests.

## Discussion

Planet Earth is home to more than 6,000 species of mammals^31^, 300,000 species of plants^32^, 5,000,000 species of insects^33^ and an unknown number of species of unicellular eukaryotes, bacteria and archaea^34,35^. Inferring the phylogenetic relationships between the biological sequences of these organisms provides a foundation upon which we can study evolution and molecular diversity, and enables us to understand and transfer biological information between organisms. It is likely that a substantial fraction of all known species on Earth will have a representative genome in the coming decades, but methods to enable analysis at this scale of data do not yet exist. Here, we present a major advancement to OrthoFinder that substantially improves the scalability and accuracy of the method. We show that it is possible to rapidly assign gene sets from large numbers of species to a phylogenetically partitioned and structured database of biological sequences in near-linear time. We also show that using phylogenetic delimitation improves the inference of orthogroups, enhancing the accuracy of OrthoFinder and extending its performance advantage over comparator methods. OrthoFinder remains an easy-to-use, fast, accurate and fully phylogenetic orthology inference software tool. OrthoFinder also continues to be one of the few tools to provide a wide range of output information including gene duplications, gene trees, sequence alignments and single-copy ortholog sequences, allowing downstream analysis.

In the analysis presented here, OrthoFinder and FastOMA were the only orthology inference tools capable of analyzing datasets comprising thousands of genomes. Despite differences in implementation, the two methods share several methodological features and workflow principles. Both methods rely on multiple sequence alignments and gene tree inference to identify orthologs from orthogroups. Each method also uses a species tree overlap method for identifying gene duplication events and identifies hierarchical orthogroups at each internal branch in the species tree. However, both methods differ substantially in approaches to scalability and requirements. OrthoFinder has been designed to run ab initio, requiring only a set of proteomes from the user. By contrast, FastOMA requires a user-specified species tree and a copy of the OMA database that contains a set of precomputed hierarchical orthogroups. In terms of scalability and accuracy, OrthoFinder outperformed FastOMA on all tests presented here and also provided a larger array of outputs, including gene trees and comparative genomic statistics.

The updated OrthoFinder method is now capable of analyzing thousands of species using a two-step process. Although this process requires an additional step for the user, the only input required is still the set amino acid sequences corresponding to the protein-coding genes for the species of interest. The default parameters for OrthoFinder have been optimized for speed, accuracy and scalability and enable the combined analysis of thousands of species on commonly available computing resources. OrthoFinder also retains its customizability for expert users, and intermediate steps in the algorithm (such as alignment or tree inference) can be substituted with alternative methods should the user wish. Currently OrthoFinder is limited to thousands of species and addressing methodological and algorithmic limitations surrounding computation resource requirements are paramount for future development. This upgrade to OrthoFinder provides and important step toward the goal of being able to provide high-accuracy phylogenetic orthology inference for all species on Earth.

## Methods

### Implementation

OrthoFinder is a Python application (version ≥3.11), which relies on several external dependencies. A default OrthoFinder run requires DIAMOND to compare protein sequences, FAMSA to align orthogroup sequences and FastTree to produce gene trees. OrthoFinder uses multiprocessing to speed up these steps by computing results in parallel. For internal steps, OrthoFinder also requires the following well-used libraries: Numpy, Scipy, Biopython, Rich and Scikit-learn. OrthoFinder includes Linux versions of the binaries for all dependencies; however, we recommend that users run OrthoFinder within a conda environment regardless of operating system. Full instructions on the installation and implementation of OrthoFinder are available via GitHub at https://github.com/OrthoFinder/OrthoFinder. OrthoFinder now has a test module, where we conduct integration tests comparing predefined expected outputs with the results of OrthoFinder. We conduct these tests for several features, including ortholog calling and gene tree-species tree reconciliation.

Full details are provided in Supplementary Section 2.

### The OrthoFinder v3 enhanced scalability workflow

The scalable linear species addition framework of OrthoFinder v3 requires the results of a conventional OrthoFinder analysis of an ‘core’ set of species. For each orthogroup, OrthoFinder v3 employs the SHOOT profile algorithm^19^ to select the most representative sequences using *k*-means clustering applied to an embedding of the sequences from the length *L* multiple sequence alignment of the orthogroup in a 2×*L*-dimensional space. DIAMOND^13^ is used to assign the new genes to the core orthogroups using these profiles. Genes not assigned to any core orthogroups are set aside to be analyzed separately at a later stage. Once all genes have been assigned, gene trees are inferred. To support the analysis of larger datasets, the MAFFT multiple sequence alignment method has been replaced with the more scalable FAMSA method^36^. Users can revert back to the previous default alignment method ‘-A mafft’. Maximum likelihood tree inference of the resulting alignments is then performed using FastTree^37^ employing the ‘-fastest’ runtime option. The use of other alignment or tree inference programs is supported through use of the config.json file, although users are advised to test if they have sufficient computational resources to use more computationally expensive programs. Once all orthogroup trees are inferred, a revised species tree is then computed using ASTRAL-Pro^38^.

As mentioned above, some genes fail to be assigned to any core orthogroups during the initial assignment step. This occurs because the new species (or sets of species) being added will sometimes contain new genes that have arisen de novo and are not found in the core species set.

To resolve this, OrthoFinder performs a root to tip tree traversal to identify clades of species (minimum size 2) that had no representation in the now sparsely sampled initial core species set.

Once identified, a conventional OrthoFinder analysis is performed on the clade, and the new orthogroups are added to the revised orthogroup set.

The original OrthoFinder algorithm applied a per-species-pair gene-length normalization that accounted for both evolutionary distance and gene-length dependence in pairwise bit scores between sequences^12^. However, this normalization step requires a large number of pairwise hits between the genes in each species to fit the required correction functions. To enable the smaller set analysis above, the updated version of OrthoFinder employs an alternative normalization approach. The approach separates the sequence length bias and species divergences to obtain many independent estimates of the extent of each orthogroup. Specifically, bit scores between two sequences, *B*_qh_, are length normalized using geometric mean of the lengths of the query and hit sequences, *L*_q_ and *L*_h_: *B*_qh _= *B*_qh_/(*L*_q_*L*_h_)^1/2^. As before, normalized reciprocal best hits (RBHs) are treated as putative orthologs and are used to estimate the sequence divergence between pairs of species and on a per-orthogroup basis.

In the modified version, we calculate a set of ratios R*_XY_ for species *X* and *Y*. These estimate the expected ratio of the normalized bit score between the RBH for a pair of genes *x* and *y* from species *X* and *Y* from some orthogroup and the normalized bit score from the gene *x* to the most distant gene in that orthogroup. Let uppercase *X*, *Y* and *Z* indicate species and lowercase letters *x*, *y* and *z* indicate genes in those species. Let *x* ↔ *y* indicate *x* is an RBH of *y* with respect to the length-normalized bit scores (NBS). Note that these are similarity scores between sequences rather than distances. Let **R**_*XYZ*_ = {*B*_*xy*_/*B*_*xz*_: *x* ∊ *X*, *y* ∊ *Y*, *z* ∊ *Z*, *x* ↔ *y*, *y* ↔ *z*}. That is, for all genes in *X* that have an RBH in both *Y* and *Z*, we calculate the ratio of the NBS between these pairs. The distribution of values in **R**_*XYZ*_ gives the statistics for calculating, given a gene *x* and its ortholog *y* with NBS *B*_*xy*_, the range of expected NBS for its ortholog in *Z*. This distribution of ratios applies across all orthogroups.

For each triple of species *X*, *Y* and *Z*, let R*_XYZ_ be the 90th percentile of ratios in **R**_XYZ_ and let ${{R}^{* }}_{XZ}=\min ({R* }_{XYZ}\forall Y\}$.

Given an RBH *x*↔*z* with NBS *B*_*xz*_, it is expected that 90% of orthologs in species *Y* will have an NBS greater than *B*_*xz*_R*_XYZ_ and that the most distant ortholog in the orthogroup will be greater than *B*_*xz*_R*_XZ_.

If a gene has RBHs in multiple species, then it provides multiple independent estimates of the cutoff for the most distant sequence in the orthogroup. Likewise, if multiple genes from a (currently undetermined) orthogroup obtain one or more RBH, then these will each provide multiple independent estimates of the extent of the orthogroup. These estimates are used to construct a graph such that a pair of sequences is connected within the graph if the NBS between those sequences fall within that gene-specific and species-specific cutoff. As such, this graph attempts to connect as densely as possible with edges all sequences within the same orthogroup while having as few edges as possible between sequences in different orthogroups. To do this, for every gene, *x*, its RBH are identified. The gene is connected in the graph to all its RBH and any other gene *y* if *B*_*xy*_ > *B*_*xz*_R*_XZ_. Here, *x* ~ *y* denotes that genes *x* and *y* are connected by an edge in the graph. That is, the set of nodes for the graph corresponds to the set of all genes, and the edge set for the graph is given by $\{x \sim y|\exists z,x\leftrightarrow z,{B}_{{x}{y}} > {B}_{{x}{z}}{R}^{* }XZ\}$.

The 90th percentile is used so that the process is intentionally over-inclusive when gathering genes into putative orthogroups, as the later phylogenetic delineation of orthogroups provides a more rigorous method of assessing orthogroup inclusion or exclusion. All that is required is that all possible members of the orthogroup are included in one tree. For this, it is not a problem if the tree covers multiple orthogroups.

As before, the MCL algorithm^39^ is applied to identify the sets of genes in the graph best satisfying the putative shared orthogroup memberships encoded in the edges of this graph. An inflation factor of 1.2 is used, as this is the value that maximizes recall in our previous parameter training^16^. While this value of inflation leads to fairly large clusters that can merge orthogroups and harm precision, our phylogenetic delineation algorithm corrects these errors. As with the core orthogroups, the resulting gene trees of the putative orthogroups are analyzed to phylogenetically resolve the true extent of each orthogroup (Fig. 1). The noncore orthogroups are subjected to phylogenetic analysis to infer rooted gene trees, and subsequently these rooted gene trees are analyzed to determine the full comparative analysis, including orthologs, hierarchical orthogroups and gene duplication events.

### Assessing orthogroup inference accuracy

The full details on the orthogroup benchmarking, including the calculation of the scores, and a complete method by method breakdown of the results are provided in Supplementary Section 1, and the complete set of output files generated by each method is provided in Supplementary File 1.

### Assessing scalability performance

The Ensembl^28^ rapid release dataset was accessed on 29 August 2024. Amino acid sequences for all protein-coding genes in each genome were downloaded. For species that had more than one version available, the most recent proteome file was used. The OrthoFinder v2 script ‘primary_transcript.py’ was used to extract only the longest variant for each gene, giving a dataset with a total of 1,789 species. Following collection of the Ensembl proteomes, an approximate species tree was derived in to extract a set of representative proteomes for performance testing across each orthology inference tool. The species tree was generated using orthologs of BUSCO genes^40^. In brief, the hidden Markov model provided for each BUSCO gene was used to identify high-scoring amino acid sequences in each species. The resulting hits from each species to each BUSCO gene were aligned using MAFFT, and the resulting multiple sequence alignments were concatenated. An unrooted phylogenetic tree was then inferred from this concatenated alignment using FastTree. This tree was then used as input to PDA^29^ to select sets of species in the range of 2–1,024 species such that each subsequent set contained all the species of the previous set, and that each subset maximized the sampled branch length of the inferred tree. The complete dataset of results for runtime and scalability of all methods is provided in Supplementary File 2.

### Assessing ortholog inference accuracy

OrthoFinder v3 was run on the 2022 edition of the QfO reference proteomes. We ran both OrthoFinder v3 Align (with FAMSA), and OrthoFinder v3 Linear (–core –assign). OrthoFinder v2, along with 13 other orthology inference tools, has already been run on this dataset and is available as part of the publicly available results alongside many methods. The QfO datasets consists of 78 proteomes (48 eukaryotes, 23 prokaryotes and 7 archaea). We ran OrthoFinder on these input datasets and extracted pairs of orthologous genes identified by the method. The file containing orthologous pairs was uploaded to the QfO web server for benchmarking. The QfO papers describe the methodology for benchmarking in full detail^3,30^.

### Reporting summary

Further information on research design is available in the Nature Portfolio Reporting Summary linked to this article.

## Online content

Any methods, additional references, Nature Portfolio reporting summaries, source data, extended data, supplementary information, acknowledgements, peer review information; details of author contributions and competing interests; and statements of data and code availability are available at https://doi.org/10.1038/s41592-026-03126-6.

## Supplementary information


Supplementary InformationSupplementary Figs. 1 and 2, Supplementary Section 1 ‘Orthogroup benchmarking’ with Supplementary Table 1.1 and Section 2 ‘Integration testing’ with Supplementary Figs. 2.1–2.7.
Reporting Summary
Peer Review File
Supplementary Data 1OrthoBench source data for each orthology inference method.
Supplementary Data 2Scalability data runtime and RAM source data for each orthology inference method.


## Data Availability

The QfO benchmarking was obtained from the QfO database. The OrthoBench data were obtained from the OrthoBench database, and all remaining datasets were obtained from Ensembl.

## Code availability

OrthoFinder is available via GitHub at https://github.com/OrthoFinder/OrthoFinder.
