# Author Correction: OrthoFinder: improved phylogenetic orthology inference with enhanced accuracy and scalability

**DOI:** 10.1038/s41592-026-03238-z

**Published:** 2026-08-26

**Authors:** David M. Emms, Yi Liu, Laurence Belcher, Jonathan Holmes, Steven Kelly

**Affiliations:** Department of Biology, University of Oxford, Oxford, UK

**Keywords:** Software, Genome informatics, Evolution, Evolutionary biology, Comparative genomics

Correction to: *Nature Methods* https://doi.org/10.1038/s41592-026-03126-6, published online 9 June 2026.

In the version of the article initially published, an incorrect version of Fig. 2 was included. The correct version of Fig. 2 has now been added to the HTML and PDF versions of the article.

