# Supplementary material for: OrthoFinder: improved phylogenetic orthology inference with enhanced accuracy and scalability
Source: Nat Methods. 2026 Jun 9;23(7):1327–33. doi: 10.1038/s41592-026-03126-6 (PMC13346109; doi:10.1038/s41592-026-03126-6)
Supplement: Supplementary file 1 — Supplementary Figs. 1 and 2, Supplementary Section 1 ‘Orthogroup benchmarking’ with Supplementary Table 1.1 and Section 2 ‘Integration testing’ with Supplementary Figs. 2.1–2.7. [file 41592_2026_3126_MOESM1_ESM.pdf]

nature methods

Supplementary information https://doi.org/10.1038/s41592-026-03126-6

OrthoFinder: improved phylogenetic orthology inference with enhanced accuracy and scalability

In the format provided by the authors and unedited

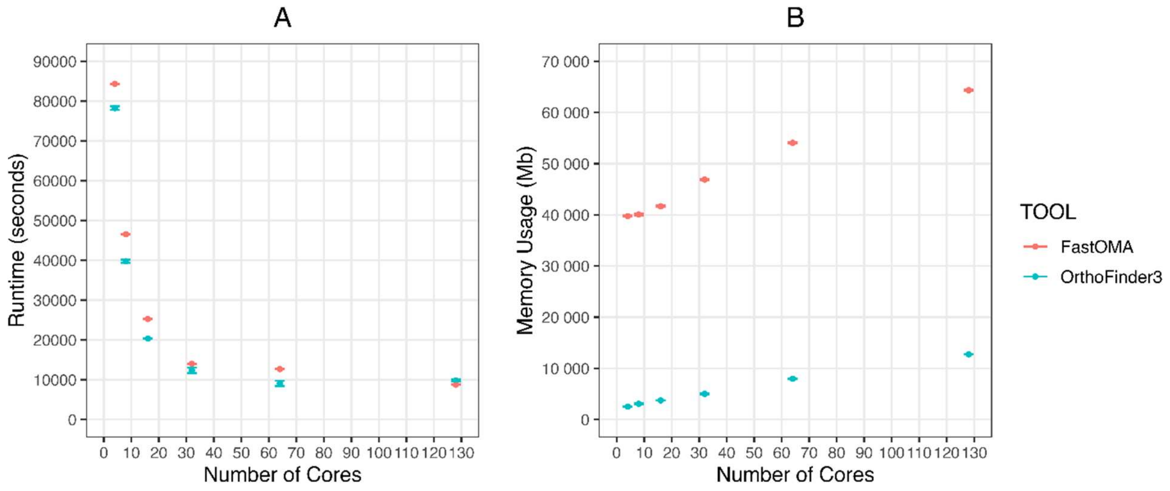

Supplementary Figure 1 : A comparison of runtime and peak memory usage for orthogroup inference on a 64 proteomes dataset by OrthoFinder v3 Linear and FastOMA across a range of threads(From 4 to 128). A) Time (log scale) and (B) peak RAM usage required to run the orthology prediction tool across a range of threads. OrthoFinder v3 first ran a core of 16 proteomes before ‘assigning’ the remaining 48 proteomes to this core, with the runtime being the sum of the ‘core’ and ‘assign’ runs.

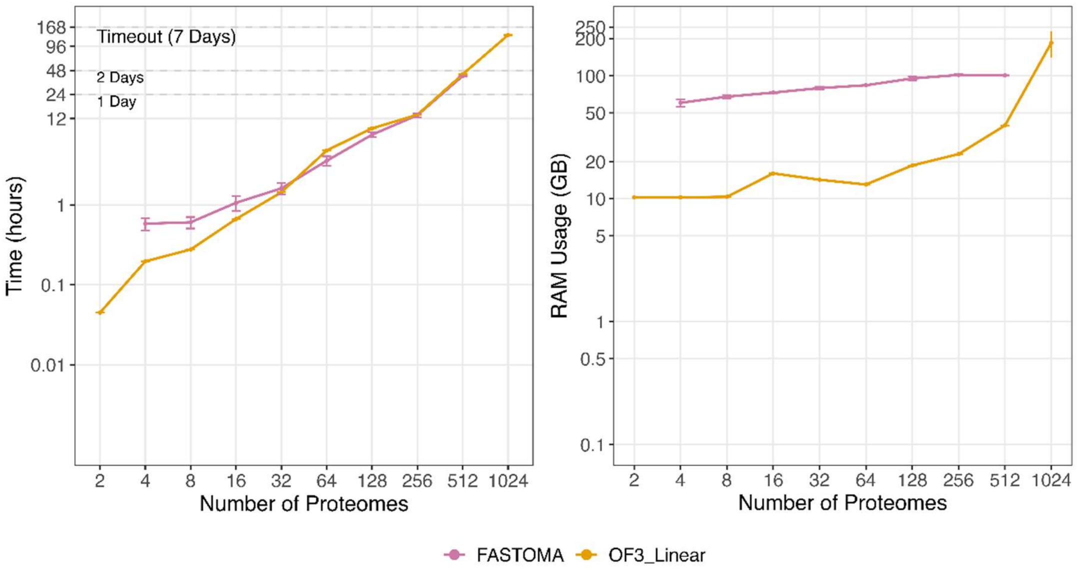

Supplementary Figure 2. A comparison of runtime and peak memory usage for orthogroup inference by OrthoFinder v3 Linear and FastOMA. (A) Time (log scale) and (B) peak RAM usage required to run the different orthology prediction tools across increasing numbers of input proteomes (from 2 to 1024) in triplicate. When the number of proteomes is <=64, OrthoFinder v3 was run using the OrthoFinder v2 workflow. When the number of proteomes is >64, OrthoFinder v3 is used to 'assign' the remaining proteome to the core set of 64 proteomes. Runtime for OrthoFinder v3 with >64 proteomes is the sum of runtime of the 'core' run and runtime of the 'assign' run.

Supplementary S1: Orthogroup Benchmarking

The aim of orthogroup benchmarking is to assess the accuracy of the set of orthogroups predicted by an orthology inference tool. We do this by comparing a set of predicted orthogroups to the 'OrthoBench' reference set of expert-curated orthogroups (Emms et al. 2020).

The OrthoBench data benchmarking problem can be mathematically summarised as follows.

Let $\mathcal{G} = \{g_1, g_2, \ \dots, g_N\}, N \in \mathbb{N}$ be a countable finite set of genes of interest. Given two clustering of $\mathrm{R} = \{R_1, R_2, \ \dots, R_S\}$ with $S \in \mathbb{N}$, $\bigcup_{i=1}^{S} R_i \subseteq \mathcal{G}$ and $\mathrm{P} = \{P_1, P_2, \ \dots, P_L\}$ with $L \in \mathbb{N}$, $\bigcup_{j=1}^{L} P_i \subseteq \mathcal{G}$), they represent the reference orthogroups (RefOGs) and predicted orthogroups (PredOGs), respectively.

The partitions in RefOGs and PredOGs are pairwise disjoint, i.e., $\bigcap_{i=1}^{S} R_i = \bigcap_{j=1}^{L} P_i = \emptyset$. The goal of benchmarking is to quantify the difference between the two clusterings R and P. Here, we proposed seven measures to achieve this goal.

1. Missing RefOGs (%)

   Summary: Missing RefOGs (%) measures the percentage of reference orthogroups that have no genes recovered in any predicted orthogroup.

   In certain methods, it is possible for no genes to be identified in a partition derived from reference orthogroups. This means that $\exists M \in \mathbb{N}, M < S$, $\sum_{i=0}^{M} \sum_{j=0}^{L} \left| R_i \cap P_j \right| = 0$. In other words, $\exists \mathbf{MR} \subseteq \mathbf{R}$, such that
   $$\mathbf{MR} = \left\{ R_i \in \mathrm{R} \;\middle|\; \forall P_j \in \mathrm{P}, R_i \cap P_j = \emptyset \right\}$$
   With this definition, the missing RefOGs (%) is given by
   $$100 \times \frac{|\mathbf{MR}|}{S}$$
   where $|\mathbf{MR}| = M$ counts the number of missing RefOGs.
2. Missing Genes (%)

   Summary: Missing Genes (%) measures the percentage of all genes in reference orthogroups that are not found in any predicted orthogroup.

As for the missing genes, we can first define a missing genes set for each RefOG, namely

$$\mathbf{MG}(R_i) = \{g \in R_i \mid \forall P_j \in \mathbf{P}, g \notin P_j\}$$

Then the missing genes measure can be computed as

$$100 \times \frac{\sum_{i=0}^{S} |\mathbf{MG}(R_i)|}{S}$$

where $|\mathbf{MG}(R_i)|$ is the size of the missing genes set of a RefOG R_i.

3. RefOG Fusions (%)

   Summary: RefOG Fusions (%) measures the percentage of reference orthogroups that have instances where they are merged into the same predicted orthogroup as another reference orthogroup. This indicates over-clustering of orthogroups.

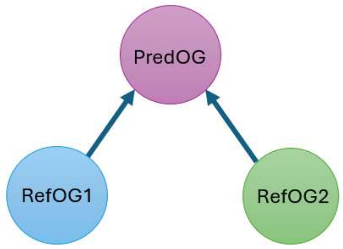

   This measure quantifies the number of RefOGs that are associated with a shared PredOG. It captures situations where multiple RefOGs overlap with the same PredOG, we define

$$\mathbf{R}'(P_j) = \{R_i \in \mathbf{R} \mid R_i \cap P_j \neq \emptyset\}$$

   which represents a set of RefOGs that have non-empty intersection with P_j. Then $\forall P_j \in \mathbf{P}$, the union of the $\mathbf{R}'(P_j)$ when $|\mathbf{R}'(P_j)| > 1$ gives the all the RefOGs that have shared PredOGs, can be rewritten as

$$\mathbf{R}' = \bigcup_{\forall P_j \in \mathbf{P}: \, |\mathbf{R}'(P_j)| > 1} \mathbf{R}'(P_j)$$

   Then the fusion score can be obtained by dividing the cardinality of $\mathbf{R}'$ (i.e., the number of fused RefOGs) over all the total number of RefOGs, namely

$$100 \times \frac{|\mathbf{R}'|}{S}$$

4. RefOG Fissions (%)

   Summary: RefOG Fissions (%) measures the percentage of reference orthogroups that have instances where they are split across multiple predicted orthogroups. This indicates over-fragmentation of orthogroups.

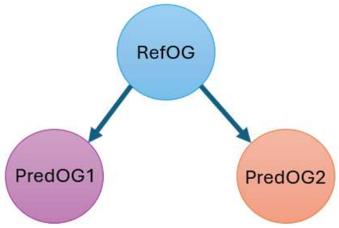

As the opposite of Fusion, Fission captures percentage of RefOGs are split into multiple PredOGs, which can be defined as follows:

$$100 \times \frac{\sum_{i=0}^{S} \mathbf{1}_{|\mathbf{P}'(R_i)|>1}}{S}$$

where

$$\mathbf{P}'(R_i) = \{P_j \in \mathbf{P} \mid R_i \cap P_j \neq \emptyset\}$$

Represents the set of PredOGs that overlap with a given RefOG R_i, and the indicator function evaluates to 1 only when $|\mathbf{P}'(R_i)| > 1$, i.e., R_i overlaps with more than one PredOG, indicating a fission.

5. Recall

Summary: Recall measures the proportion of genes in each reference orthogroup that are recovered in predicted orthogroups, computed as a weighted average across overlaps and then averaged over all reference orthogroups with size-based weighting.

Recall and Missing Genes (%) are complementary metrics that both evaluate the recovery of genes from reference orthogroups. Recall emphasizes the proportion of successfully identified genes, while Missing Genes (%) highlights gene loss.

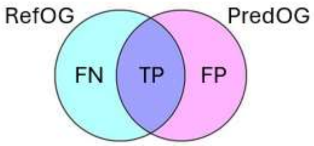

As a well-known measure, recall measures how well a method identifies all relevant instances (true positives) from the total number of actual relevant instances, which is defined as follows:

$$RC = \frac{TP}{TP + FN}$$

where TP stands for true positives, FN stands for false negatives.

Following this definition, the recall between the ith RefOG and the jth PrefOG is given by

$$rc_{ij} = \frac{|R_i \cap P_j|}{|R_i|}$$

where $|R_i \cap P_j|$ is the size of the intersection between RefOG R_i and PredOG P_j. $|R_i|$ is the size of RefOG R_i.

Since a RefOG can be split into multiple PredOGs, in this study, we favor the weighted average version of the recall score to fully capture the relationship between the RefOGs and the PredOGs. For each RefOG, we can define the weighted average recall as follows:

$$RC_i = \frac{\sum_{j=0}^{L} |R_i \cap P_j| * rc_{ij}}{\sum_{j=0}^{C} |R_i \cap P_j|}$$

Under this context, $|R_i \cap P_j|$ is chosen as the weight.

To obtain the total average scores for all RefOGs, we can apply the same idea across all RefOGs to get the total weighted average recall score, namely,

$$\mathbf{RC} = \frac{\sum_{i=0}^{S} |R_i| * RC_i}{\sum_{i=0}^{S} |R_i|}$$

Here, the size of each RefOG $|R_i|$ is used as the weight.

6. Precision

Summary: Precision measures the proportion of genes in each predicted orthogroup that correctly match genes from the corresponding reference orthogroup, computed through overlap-based weighting and averaged across all reference orthogroups.

For precision, it's general definition is given by the following expression,

$$PS = \frac{TP}{TP + FP}$$

where FP stands for false positive. Then, the precision between the ith RefOG and the jth PrefOG is given by

$$ps_{ij} = \frac{|R_i \cap P_j|}{|P_j|}$$

Similarly we can define the weighted average precision for each RefOG and the total weighted average precision for all RefOGs as

$$PS_i = \frac{\sum_{j=0}^{L} |R_i \cap P_j| * ps_{ij}}{\sum_{j=0}^{C} |R_i \cap P_j|}$$

and

$$\mathbf{PS} = \frac{\sum_{i=0}^{S} |R_i| * PS_i}{\sum_{i=0}^{S} |R_i|}$$

respectively.

7. Entropy

Summary: Entropy measures the fragmentation of each reference orthogroup by computing the Shannon entropy of how its genes are distributed across predicted orthogroups (including an extra category for missing genes), with the final score being the size-weighted average of normalized entropy values across

all reference orthogroups — higher values indicate greater dispersion of genes across predictions.

Both RefOG Fission (%) and Entropy assess how genes from a reference orthogroup are distributed across predicted orthogroups; Fission provides a coarse, binary indicator of splitting events, whereas Entropy offers a fine-grained, Shannon entropy–based measure of fragmentation severity.

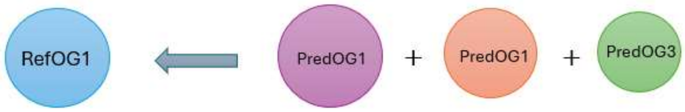

Since each RefOG can be split into multiple PredOGs, we introduce entropy as a measure to capture how spread out such effect is.
For each cluster in $\mathbf{R}$, each gene can be assigned a different label if it is classified into different PredOGs. Therefore, we define the set

$$\mathbf{C}(R_i) = \{P_j \in \mathbf{P} \mid R_i \cap P_j \neq \emptyset\} \bigcup \left\{ P_m \mid g \in R_i, g \notin \cup_{P_j \in \mathbf{P}} P_j \right\}$$

to be the possible labels (PredOGs) assigned to the genes in R_i, which includes a label P_m assigned to all missing genes for R_i. With this definition, we can obtain the probability of a gene in R_i being classified into P_j

$$\mathbb{P}(P_j \mid R_i) = \frac{|R_i \cap P_j|}{|R_i|}$$

and P_m,

$$\mathbb{P}(P_m \mid R_i) = \frac{|P_m|}{|R_i|}$$

where

$$|P_m| = |R_i| - \sum_{P_j \in \mathbf{P}} \left|R_i \bigcap P_j\right|$$

According to the Shannon entropy, the entropy of a RefOG R_i can be defined as

$$H_i = -\mathbb{P}(P_m \mid R_i) \log_2 \mathbb{P}(P_m \mid R_i) - \sum_{P_j \in \mathbf{P} | R_i \cap P_j \neq \emptyset} \mathbb{P}(P_j \mid R_i) \log_2 \mathbb{P}(P_j \mid R_i)$$

Here, H_i is unbounded, we can normalize it by dividing it by the maximum possible entropy a RefOG R_i can achieve. The maximum entropy occurs when all genes in R_i are equally distributed across all possible classifications.

$$NH_i = \frac{H_i}{\log_2 |R_i|}$$

Here, the normalised entropy for each RefOG R_i is denoted by NH_i, which is bounded between 0 and 1. If and only if all genes in R_i are assigned to a single classification, $NH_i = 0$.

Similar to recall and precision, we can compute the weighted average of normalised entropy across all RefOGs, weighted by the size of each RefOG $|R_i|$. This is given by,

$$\mathbf{H} = \frac{\sum_{i=0}^{S} |R_i| * NH_i}{\sum_{i=0}^{S} |R_i|}$$

$\mathbf{H}$ has the same interpretation as NH_i, but aggregated over all RefOGs. A small value indicates better performance, as it reflects lower uncertainty or more concentrated classifications for RefOGs, whereas a higher value indicates worse performance, as it reflects higher uncertainty or more scattered classifications. The interpretation is similar to NH_i. The smaller the value of $\mathbf{H}$ is, the better the methods are.

8. Rank Score

Summary: Rank Score quantifies the overall performance of a method by ranking it relative to others on each evaluation measure, then averaging those ranks across all measures — lower scores indicate better overall performance.

To quantify the performance of each method, we introduce the rank score in this study, which takes the rank of each method against each measure mentioned above, then average the rank for each method across all measures.

Let X be a random variable that takes elements from a countable set of methods $\mathcal{X} = \{x_1, x_2, \dots x_q\}, q \in \mathbb{N}$ if the number of methods. We can define a score function $s: X \rightarrow \mathbb{R}$, such that $\forall x_i \in \mathcal{X}, s(x_i) \in \mathbb{R}$.

With the score function, a rank function $\rho: X \rightarrow \{1, 2, \dots q\}$ which assigns a rank to each element $x_i \in \mathcal{X}$ based on its position in the sorted order can be expressed as follows:

$$\rho(x_i) = \left|\{x_j \in \mathcal{X} \mid s(x_j) \leq s(x_i)\}\right| + 1$$

here $\rho(x_i)$ counts the number of elements x_j with scores less than $s(x_i)$ then adds 1 to give the rank.

Now let $\mathcal{M} = \{m_1, m_2, \dots, m_p\}$ be a countable set of measures, we can define the rank score for each method x_i against each measure m_i as

$$rs(x_i) = \{\rho(x_i \mid m_j) \mid m_j \in \mathcal{M}\}$$

where $\rho(x_i|m_i)$ represents the rank of x_i based on the measure m_j.

The final average rank score for method x_i can then be given by

$$RS(x_i) = \frac{1}{p}\sum_{j=0}^{p} \rho(x_i \mid m_j)$$

where $p = |\mathcal{M}|$ is the number of measures.

In this study, we have seven measures and 13 methods, plug in those number of into the measure definition and the rank function we can obtain the table and figure shown below. They both illustrates the performance of each method using different measures. The last column in the table gives the average rank score for each method across all measures, so as the last plot in the figure.

Supplementary Table 1.1. Methods scores for each Orthogroup benchmarking method across multiple tools.

Methods	Missing RefOGs (%)	Missing Genes (%)	RefOG Fusions (%)	RefOG Fissions (%)	Recall	Precision	Entropy	F1-score	Rank Score
OF3_Align_ST	0	2.533	2.857	32.857	84.112	82.94	0.097	78.492	2.625
OF3_DB	0	2.639	2.857	32.857	83.557	84.667	0.1	79.83	2.75
OF3_Linear	0	1.266	2.857	35.714	80.499	81.374	0.118	75.071	3.5
OF2_DB	0	2.533	2.857	35.714	78.686	83.268	0.131	74.524	3.625
OF2_Align_ST	0	2.533	2.857	35.714	78.686	83.268	0.131	74.524	3.625
Broccoli	0	3.113	2.857	27.143	75.572	80.425	0.131	70.912	4.875
SP_sens	0	5.805	2.857	35.714	70.81	85.864	0.18	68.911	5.625
OrthoHMM	0	3.166	2.857	31.429	76.183	75.725	0.137	66.395	6
SP_def	0	7.282	2.857	38.571	70.543	86.016	0.175	68.627	6.5
OrthoMCL	0	7.018	2.857	35.714	61.562	89.318	0.244	62.847	6.5
SP_fast	0	7.652	2.857	40	69.92	86.394	0.18	68.512	7
ProteinOrtho	0	16.201	2.857	65.714	37.355	95.814	0.363	47.508	9.125
Hieranoid	1.429	15.567	2.857	41.429	54.845	94.586	0.277	61.102	9.875
FastOMA	1.429	9.868	10	47.143	58.927	80.46	0.262	54.91	12.625

Supplementary S2: Integration Testing

1. Integration testing

Integration testing evaluates the successful application of modules and functions to test cases. To do this, we established a set of test cases for OrthoFinder testing a range of core functionality for successful execution. 10 test cases were developed covering the core and the assign workflow. For each of the core and assign workflow, 5 tests were performed: The identification of hierarchical orthogroups, the correctly calling of duplication events, the correct calling of orthologs, gene-tree-species-tree reconciliation, and generating correct comparative genomics statistics.

1.1 Generating ground truth data

In order to assess the successful execution of each OrthoFinder functionality a ground truth was generated for each test case. These test cases were manually curated and the expected values mapped. To generate these test cases, simulated orthogroups were developed from a simple bifurcating species tree, containing 4 species for core and adding an additional species for assign. For each test a simple gene tree was designed using the species tree template in figure 1.

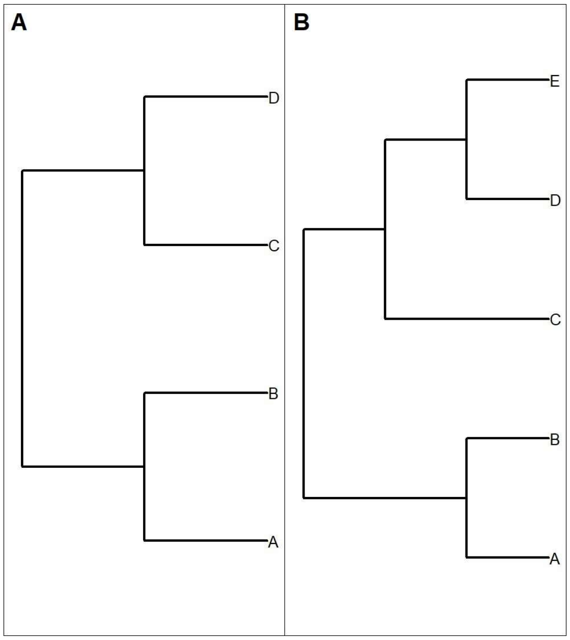

Supplementary Figure 2.1. A simple species tree used for generating simulated orthogroups for integration testing for core (A) and assign runs (B).

Sequences for each test case were generated using IQ-tree AliSim from the manually curated gene trees. Randomised amino acid sequences (of length 75-500) were used to seed the AliSim simulation, avoiding the possibility of crossover in BLAST hits between orthogroups. This ensures that no test case would impact the results of another test case in an unpredictable way ensuring data integrity. These generated sequences were then labelled with unique identifiers and packaged into proteome files.

We designed the test module to operate in two stages. The first stage systematically evaluates di erent combinations of options available in OrthoFinder. This approach serves two purposes:

(1) it ensures broader coverage of the package's functionality, and

(2) it verifies that OrthoFinder can be executed successfully without runtime errors.

The second stage focuses on accuracy testing. This phase requires two consecutive OrthoFinder runs on a curated dataset. First, OrthoFinder is run on the Core species set using a user-provided species tree. Then a second run is performed in core/assign mode, incorporating additional species into the Core set. In this stage, we assess whether OrthoFinder produces the expected outputs, including Orthogroups, Phylogenetic Hierarchical Orthogroups, Orthologues, Gene Duplication events, Resolved Gene Trees, and summary statistics.

The test suite directly calls relevant OrthoFinder functions and processes the intermediate data files produced by OrthoFinder or its external dependencies to regenerate its expected outputs. This approach not only increases confidence in the scientific accuracy of the software but also contributes to overall code coverage. Additional tests were developed to ensure that this coverage was maximised including; single copy orthogroups, unassigned genes, large orthogroups for SHOOT profiling etc.

2. Inputs and Expected Outcomes – Core

Inputs were prepared as ground truths trees where we expect a particular outcome through manual curation. These expected outcomes were then embedded in functions which were used to test the output of OrthoFinder to test successful functionality.

2.1 Hierarchal Orthogroups

An Orthogroup is defined as a set of genes descended from a single gene in the last common ancestor of all species considered. This can include genes lost or genes gained through duplication. OrthoFinder infers orthogroups at each hierarchical level from the species tree, i.e those genes that are present in a subset of species under consideration. Ancient duplications which predate the species tree may bring together genes into orthogroups where the origin may have been from several genes rather than a single gene. OrthoFinder aims to split gene trees into revised phylogenetically defined orthogroups, this is then preformed at each node of the species tree.

2.1.1 Hierarchal Orthogroups : Core

The species tree (Figure 2A) contains 3 nodes (N0, N1 and N2) for each hierarchical level. The test case (Figure 2B) shows a duplication on node N0 which maps to the root of the species tree. For correct delineation, it is expected that this orthogroup will be split into two parts. The expected outcome is the creation of two orthogroups one containing only the genes HOG_A_1, HOG_B_1, HOG_C_1, HOG_D_1 and a second containing only HOG_A_2, HOG_B_2, HOG_C_2, HOG_D_2. The species tree (Figure 2A) also contains multiple further nodes N1 and N2. For each hierarchical level of the species tree we would then expect further delineation. At N1 hierarchical level the genes HOG_A_1, HOG_B_1, HOG_A_2 and HOG_B_2 would be present with HOG_C_1, HOG_D_1, HOG_C_2 and HOG_D_2 at the N2 hierarchical level.

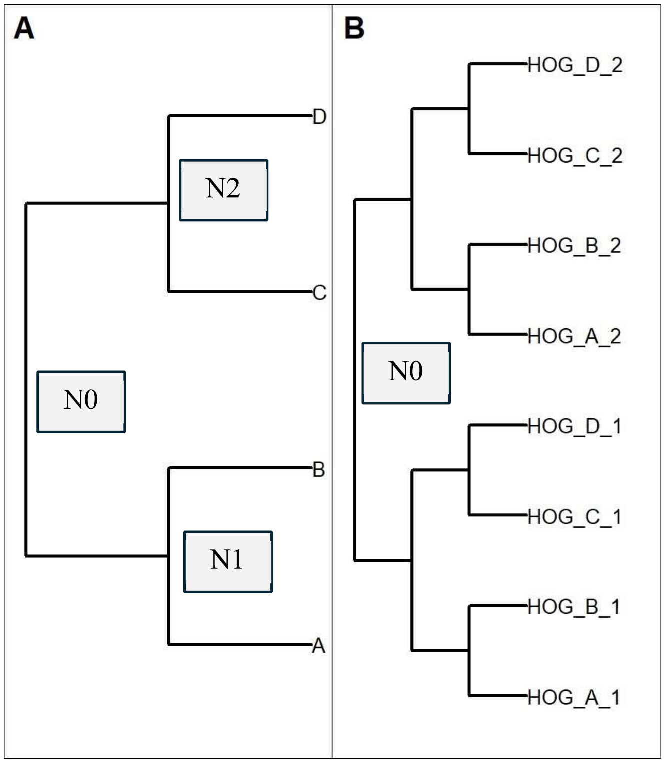

Supplementary Figure 2.2. The test case for correct delineation of an Orthogroup into a hierarchical Orthogroup. A) shows the species tree B) shows the Orthogroup test case.

2.1.2 Hierarchal Orthogroup test : Assign

To test the --assign mode of OrthoFinder a similar test case to the —core test for successful hierarchical Orthogroup assignment (Figure 2) was performed. In this case the species tree topology (Figure 3A) was again duplicated at the root node to produce a gene tree with 2 genes per species. The core run was performed using only species A, B, C and D (Figure 2A), with the remaining genes E assigned to this core tree. The gene tree used to test the successful definition during assign would be split at the root N0 node (Figure 3) producing two orthogroups containing all species A to E, with the first containing gene 1 and the second containing gene 2. Additionally, the phylogenetically hierarchical orthogroups at N1 contains two outgroups A_1, B_1 and A2_B2, N2 containing C_1, D_1, E_1 and C_2, D_2, E_2 finally N3 contains D_1, E_1 and D_2, E_2.

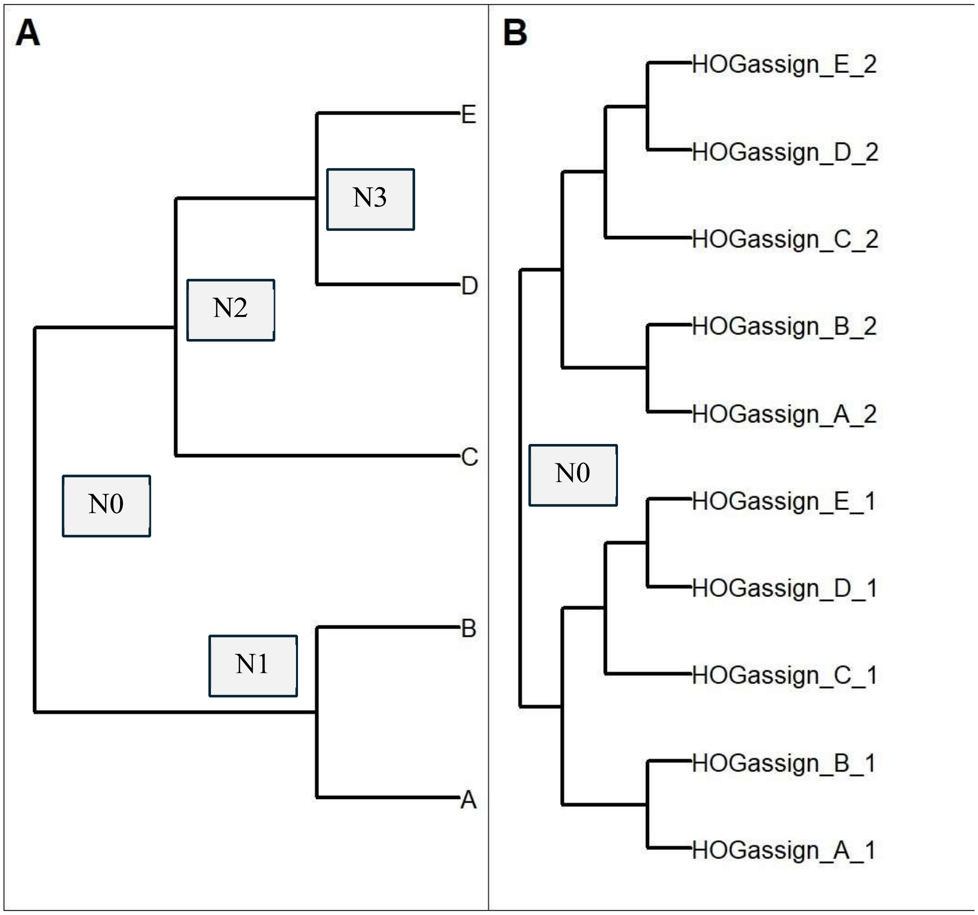

Supplementary Figure 2.3. The test case for correct delineation of an Orthogroup into a hierarchical Orthogroup during an --assign run. A) shows the species tree B) shows the Orthogroup test case.

2.2 Gene duplication

Duplications events are an important evolutionary mechanism for increasing the available pool of genetic information, providing the potential for new genes to arise. OrthoFinder aims to map these duplications to nodes of a gene tree through a duplication-loss-coalescent algorithm. In this case child nodes are described as duplication if the set of species below the child nodes overlap, otherwise these are classed as speciation events.

2.2.1 Duplication test : Core

OrthoFinder classifies duplication as occurring in three possible positions, terminal, non-terminal and root. As root duplication identification has been tested in section 2.1. A test case containing terminal and non-terminal duplications was curated (Figure 4). In this case, a gene begins at the root of the species tree (Figure 4A) with speciation event at N0 creating genes D_1 and C_1, followed by a non-terminal duplication event at N2, speciation at N3 and N6 and finally subsequence terminal duplication events at N4, N5, N7 and N8.

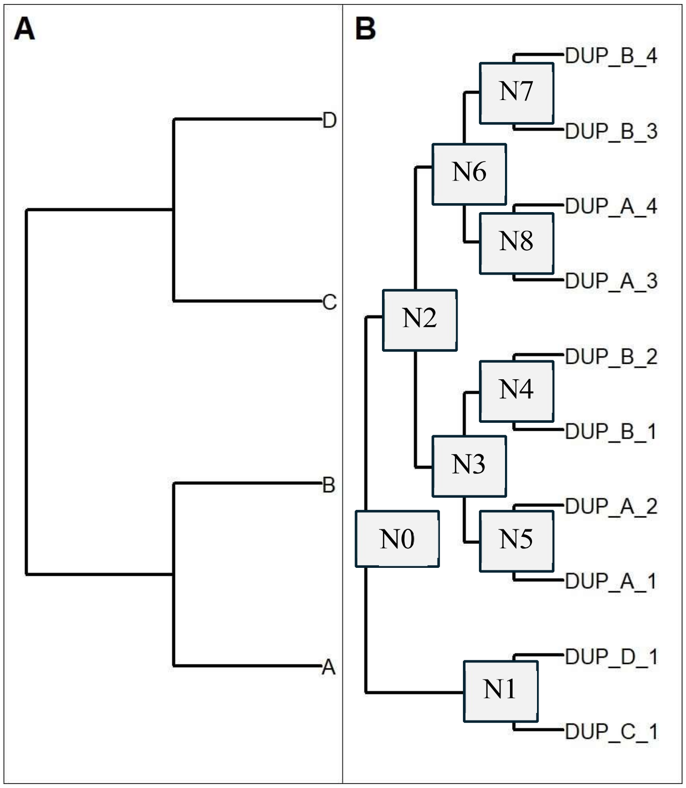

Supplementary Figure 2.4. The test case for correct delineation of an Orthogroup into a hierarchical Orthogroup. A) shows the species tree B) shows the Orthogroup test case.

2.2.2 Duplication test : Assign

As with the core duplication test, duplications are assigned to nodes on a species tree which contain two or more species on child branches. The species tree for assign which adds species E as a child of species C (Figure 5A) compared to the core species tree (Figure 4A). Non-terminal duplications of each species pair: (A,B) and (D,E) were created at the terminal ends with an additional terminal duplication for species C. Therefore correct duplication identification would observe non-terminal duplication at nodes N6, N1 and a terminal duplication at N5.

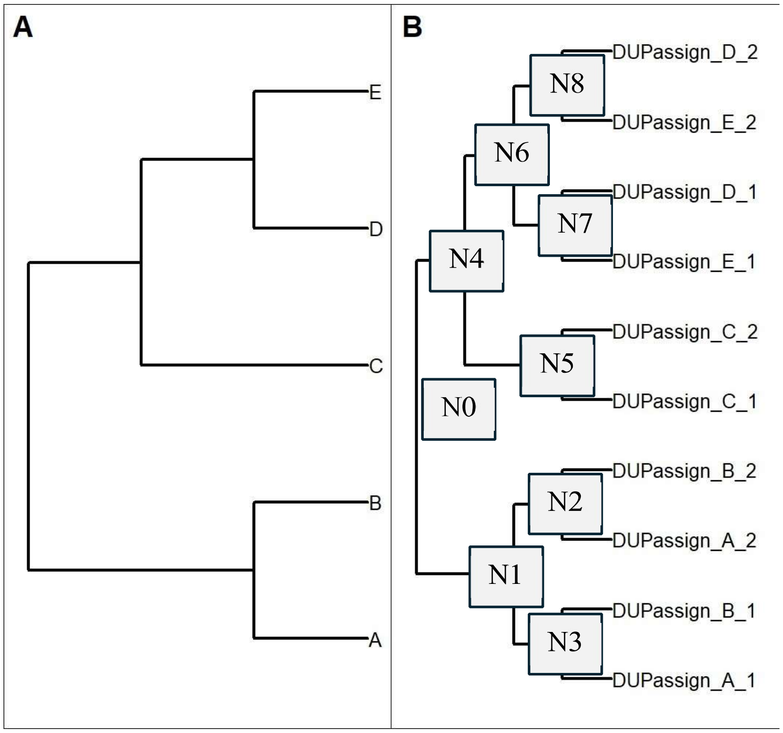

Supplementary Figure 2.5. The test case for correct delineation of an Orthogroup into a hierarchical Orthogroup. A) shows the species tree B) shows the Orthogroup test case.

2.3 Ortholog test

Ortholog calling is a core function of OrthoFinder. Orthologs are genes which have descended from a common ancestor by speciation in contrast to paralogs which diverge at a duplication node. Orthologs are therefore identified pairwise with paralogs being excluded. This is computed by taking gene trees generated from OrthoFinder orthogroups, marking duplication nodes (section 2.2) and creating a set of pairwise orthologs for each set of species under consideration.

2.3.1 Ortholog test – Core and Assign

A species tree (figure 6A) containing all proteomes (core + assign) was used to assess the correct calling of orthologs for core and assign, using a gene tree containing sets of orthologs (figure 6B). During the core run only species A,B,C and D are considered, in the case of the species tree the ortholog pairs for species A are: A_1, A_2 to C1, A1_A2 to D_1, A_1 to B1 and A_2 to B_2. For species B the ortholog pairs are: B_1, B_2 to C1, B1_B2 to D_1, B_1 to A1 and B_2 to A_2. For species C the ortholog pairs are: C_1 to A_1 and A_2, C_1 to B_1 and B_2, C_1 to D_1. Finally the ortholog pairs for species D are D_1 to A_1 and A_2, D_1 to B_1 and B_2, D_1 to C_1. Critically in these pairings A1 and A2 are not considered orthologs because they occur following a duplication and are therefore paralogs.

Finally for an assign run which includes the presence of proteome E (Figure 6A), the expectation is to add the orthologs of E to each other ortholog (Figure 6B). The additional pairs for species A are: A_1 and A_2 to E_1 and E_2. For species B: B_1 and B_2 to E_1 and E_2. For species C: C_1 to E_1 and E_2. Finally, species D: D_1 to E_1 and E_2. In addition to these new ortholog pairs for the existing core run proteome E also has its own set of ortholog pairs to the core proteomes these are: E_1 and E_2 to A_1 and A_2, : E_1 and E_2 to B_1 and B_2, E_1 and E_2 to C_1 and finally E_1 and E_2 to D_1. Again, it is critical that E_1 and E_2 are not orthologs as they occur following a duplication event.

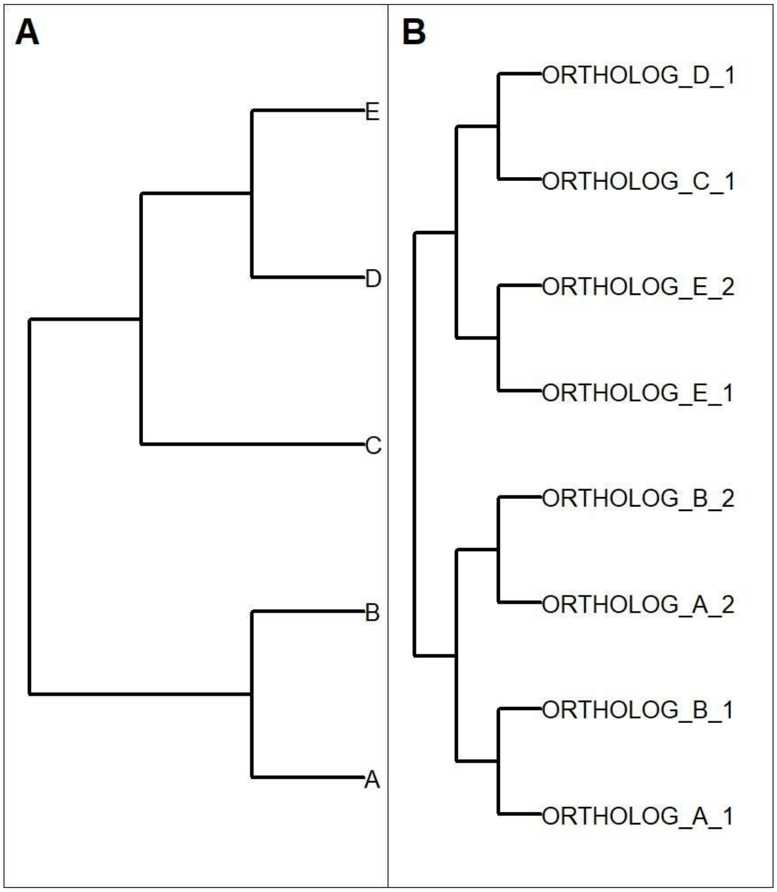

Supplementary Figure 2.6. Ortholog calling on a core + assign run showing the pairs of orthologs (B) expected for each species in the species tree (A).

2.4 Gene-tree-species-tree reconciliation test

OrthoFinder uses a novel algorithm to address incongruence between the gene tree and the species tree. The aim is to find the most parsimonious reconciliation of the gene

tree with the species tree, which will increase the accuracy of duplication and ortholog identification.

2.4.1 Gene-tree-species-tree reconciliation test – Core and Assign

Consider the species tree in Figure 7A (ignoring species E), and the gene tree in Figure 7B). The gene tree has one clade with genes from species C and D, and one clade with species from A, B, and C. A more parsimonious reconciliation would rearrange the tree to move gene RECON_C_2 to the clade containing RECON_C_1 and RECON_C_2. Therefore, our integration test asserts that genes RECON_C_2, RECON_C_1 and RECON_C_2 will form a clade in the output gene trees from OrthoFinder.

The exact algorithm that describes the criteria for reconciliation can be found in Emms et al. (2019), but we use this simple scenario for our test.

For the assign run, the two genes from species E are added. RECON_E_1 is already in a clade with species C & D, whereas RECON_E_2 is in a clade with species A & B. Our algorithm should therefore reconcile the trees to place RECON_E_2 in the clade with genes from species C, D, and E (consistent with the species tree). Our formal check is that genes RECON_C_1, RECON_E_1, RECON_C_2, RECON_E_2, RECON_D_1 should be monophyletic in the output gene tree.

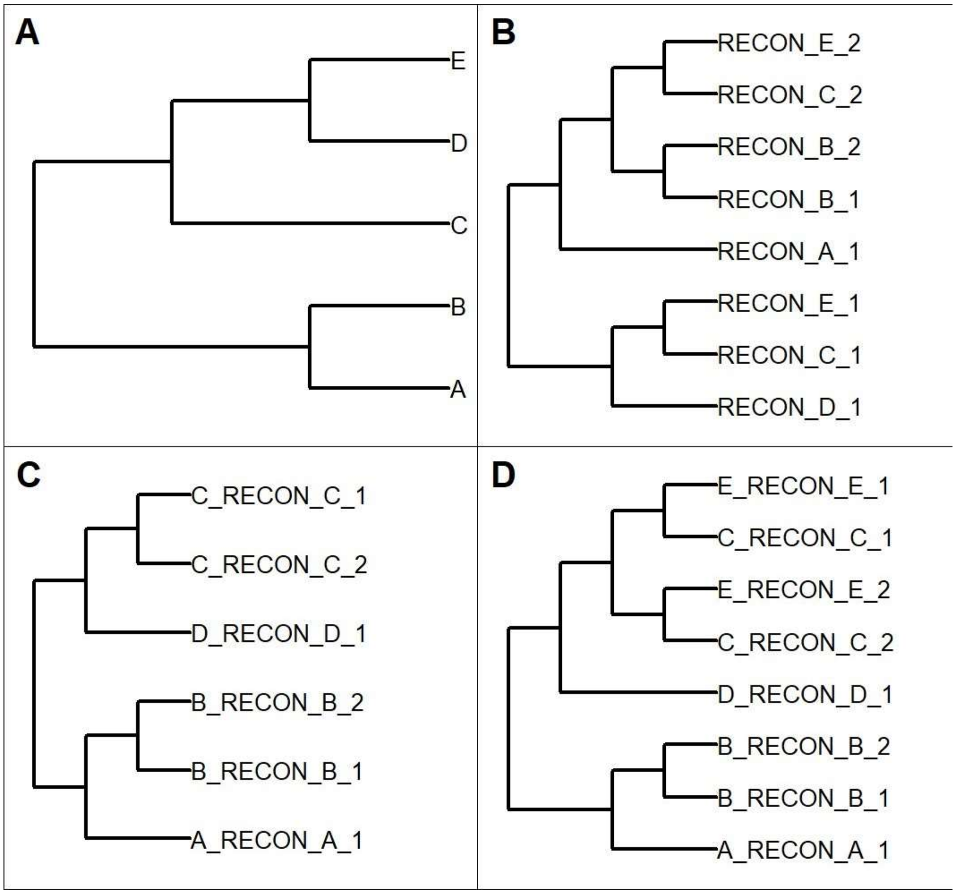

Supplementary Figure 2.7: Panel (A) shows the species tree. Panel (B) shows the input gene tree. Panel (C) shows the expected output from gene tree-species tree reconciliation for the ‘core’ run. Panel (D) shows the expected output from gene tree-species tree reconciliation for the ‘assign’ run.
