# Supplementary material for: OrthoFinder: improved phylogenetic orthology inference with enhanced accuracy and scalability
Source: Nat Methods. 2026 Jun 9;23(7):1327–33. doi: 10.1038/s41592-026-03126-6 (PMC13346109; doi:10.1038/s41592-026-03126-6)
Supplement: Supplementary file 3 — Peer Review File [file 41592_2026_3126_MOESM3_ESM.pdf]

nature portfolio

Peer Review File

OrthoFinder: Improved phylogenetic orthology inference with enhanced accuracy and scalability

Corresponding Author: Professor Steven Kelly

Version 0:

Decision Letter:

24th Sep 2025

Dear Professor Kelly,

We very much appreciate your patience when your Article, "OrthoFinder: scalable phylogenetic orthology inference for comparative genomics" is sent out for peer review. This paper has now been seen by 2 reviewers. As you will see from their comments below, although the reviewers find your work of considerable potential interest, they have raised a number of concerns. We continue to be interested in potentially publishing your manuscript in Nature Methods, but would like to evaluate your response to these concerns before we reach a final decision on publication.

We therefore invite you to revise your manuscript to address these concerns.

We are committed to providing a fair and constructive peer-review process. Do not hesitate to contact us if there are specific requests from the reviewers that you believe are technically impossible or unlikely to yield a meaningful outcome.

When revising your paper:

* include a point-by-point response to the reviewers and to any editorial suggestions

* please underline/highlight any additions to the text or areas with other significant changes to facilitate review of the revised manuscript

* address the points listed described below to conform to our open science requirements

* ensure it complies with our general format requirements as set out in our guide to authors at www.nature.com/naturemethods

* resubmit all the necessary files by using the link below to access your home page

Link Redacted

Note: This URL links to your confidential account and associated information about manuscripts you may have submitted, or that you are reviewing for us. If you wish to forward this email to co-authors, please delete this link.

We hope to receive your revised paper within 3 months. If you are substantially delayed, please let us know. In this event, we will still be happy to reconsider your paper at a later date so long as nothing similar has been accepted for publication at Nature Methods or published elsewhere.

OPEN SCIENCE REQUIREMENTS

REPORTING SUMMARY
When revising your manuscript, please update your reporting summary.

Reporting summary: https://www.nature.com/documents/nr-reporting-summary.zip

If your paper includes custom software, we also ask you to complete a supplemental reporting summary.

Software supplement: https://www.nature.com/documents/nr-software-policy.pdf

Please submit these with your revised manuscript. They will be available to reviewers to aid in their evaluation if the paper is re-reviewed. If you have any questions about the checklist, please see http://www.nature.com/authors/policies/availability.html or contact me.

Please note that these forms are dynamic ‘smart pdfs’ and must therefore be downloaded and completed in Adobe Reader. We will then flatten them for ease of use by the reviewers. If you would like to reference the guidance text as you complete the template, please access these flattened versions at http://www.nature.com/authors/policies/availability.html.

For any revision that includes light microscopy data, we ask our authors to please include a completed light microscopy reporting table [https://www.nature.com/documents/Light_microscopy_reporting_table.xlsx] to ensure the methods are described thoroughly. The table will be available to reviewers and ultimately published should the manuscript be accepted at the journal.

EXTENDED DATA FIGURES
When re-submitting your manuscript, please ensure that any supplementary figures and tables that are crucial to the manuscript’s conclusions are converted into Extended Data figures and tables to increase visibility of these data. Extended Data figures and tables are online-only (present in the online PDF and full-text HTML versions of the paper), peer-reviewed display items that provide essential background to the article but are not included in the main article due to space constraints. A maximum of ten Extended Data display items (figures and tables) is permitted.

DATA AVAILABILITY
We strongly encourage you to deposit all new data associated with the paper in a persistent repository where they can be freely and enduringly accessed. We recommend submitting the data to discipline-specific and community-recognized repositories; a list of repositories is provided here: http://www.nature.com/sdata/policies/repositories

All novel DNA and RNA sequencing data, protein sequences, genetic polymorphisms, linked genotype and phenotype data, gene expression data, macromolecular structures, and proteomics data must be deposited in a publicly accessible database, and accession codes and associated hyperlinks must be provided in the “Data Availability” section.

For papers containing bioimaging data, we strongly recommend depositing the data to Bioimage Archive (https://www.ebi.ac.uk/bioimage-archive/). Associated accession codes and hyperlinks should be provided in the "Data Availability" section.

Refer to our data policies here: https://www.nature.com/nature-research/editorial-policies/reporting-standards#availability-of-data

To further increase transparency, we encourage you to provide, in tabular form, the data underlying the graphical representations used in your figures. This is in addition to our data-deposition policy for specific types of experiments and large datasets. For readers, the source data will be made accessible directly from the figure legend. Spreadsheets can be submitted in .xls, .xlsx or .csv formats. Only one (1) file per figure is permitted: thus if there is a multi-paneled figure the source data for each panel should be clearly labeled in the csv/Excel file; alternately the data for a figure can be included in multiple, clearly labeled sheets in an Excel file. File sizes of up to 30 MB are permitted. When submitting source data files with your manuscript please select the Source Data file type and use the Title field in the File Description tab to indicate which figure the source data pertains to.

Please include a “Data availability” subsection in the Online Methods. This section should inform readers about the availability of the data used to support the conclusions of your study, including accession codes to public repositories, references to source data that may be published alongside the paper, unique identifiers such as URLs to data repository entries, or data set DOIs, and any other statement about data availability. At a minimum, you should include the following statement: “The data that support the findings of this study are available from the corresponding author upon request”, describing which data is available upon request and mentioning any restrictions on availability. If DOIs are provided, please include these in the Reference list (authors, title, publisher (repository name), identifier, year). For more guidance on how to write this section please see: http://www.nature.com/authors/policies/data/data-availability-statements-data-citations.pdf

CODE AVAILABILITY
Please include a “Code Availability” subsection in the Online Methods which details how your custom code is made available. Only in rare cases (where code is not central to the main conclusions of the paper) is the statement “available upon request” allowed (and reasons should be specified).

We request that you deposit code in a DOI-minting repository such as Zenodo, Gigantum or Code Ocean and cite the DOI in the Reference list. We also request that you use code versioning and provide a license.

For more information on our code sharing policy and requirements, please see:
https://www.nature.com/nature-research/editorial-policies/reporting-standards#availability-of-computer-code

MATERIALS AVAILABILITY
As a condition of publication in Nature Methods, authors are required to make unique materials promptly available to others without undue qualifications.

Authors reporting new chemical compounds must provide chemical structure, synthesis and characterization details. Authors reporting mutant strains and cell lines are strongly encouraged to use established public repositories.

More details about our materials availability policy can be found at https://www.nature.com/nature-portfolio/editorial-policies/reporting-standards#availability-of-materials

ORCID
Nature Methods is committed to improving transparency in authorship. As part of our efforts in this direction, we are now requesting that all authors identified as 'corresponding author' on published papers create and link their Open Researcher and Contributor Identifier (ORCID) with their account on the Manuscript Tracking System (MTS), prior to acceptance. This applies to primary research papers only. ORCID helps the scientific community achieve unambiguous attribution of all scholarly contributions. You can create and link your ORCID from the home page of the MTS by clicking on 'Modify my Springer Nature account'. For more information please visit please visit www.springernature.com/orcid.

Please do not hesitate to contact me if you have any questions or would like to discuss these revisions further. We look forward to seeing your revised manuscript and thank you for the opportunity to consider your work.

Best regards,

Lin Tang, PhD
Senior Editor
Nature Methods

Reviewers' Comments:

Reviewer #1 (Remarks to the Author):

The manuscript describes the OrthoFinder3 method for orthology inference. It builds on the highly successful OrthoFinder2 software by the same lab, which has garnered over 6000 citations since 2019. Input is a set of proteomes, each comprising the protein sequences encoded in the genome for a certain species. As in version 2, the output is a list of orthogroups, ortholog pairs between species, species trees etc. The main improvement over version 2 is the speed improvement (roughly a factor 13 for n = 256 proteomes) and a slightly better scaling with the n. This was achieved using a similar idea as in FastOMA (Nature Methods 2025): to run the slow O(n^2) all-vs-all similarity search needed for a first clustering of input proteins into tentative orthogroups only on a precomputed species set (FastOMA) or on small subset of the input proteomes (Orthofinder3). The proteins from the other species/proteomes are then assigned to one of the tentative orthogroups. In the next step, both methods run their magic to split the resulting tentative orthogroups up into the actual, orthogroups returned to the user.

Orthofinder3 has nearly the same performance as Orthfinder2 while being much faster. It has very similar speed as FastOMA, and these two tools are the only ones able to run on large input sets (>1000 species) in acceptable time (Fig. 4). However, OF3's accuracy according to various measures in the standardized Quest for Orthologs (QfO) benchmark is quite a bit better than that of FastOMA (Fig 5).

The methodological improvements are rather simple and reasonable and rely partly on integrating previous work such as SHOOT into OF3, but the end result, given the enormous popularity of OF2 due to its unmatched accuracy and usablility, could make OF3 with its improved speed very popular as well, if the scaling issues for memory and a few other points detailed below are addressed properly.

Major issues

1. The better speed and possibly better scaling (theoretically down to n instead of n^2) is the main selling point of OrthoFinder. However, while in the speed benchmark of FastOMA (Nature Methods 2025, Fig. 2c) one could see a clean O(n^2) scaling of OF2 and other tools and O(n) for FastOMA, the speed benchmark in the present study does not show any clean scaling at all. In fact, unexpectedly the scaling of FastOMA and OF3 for the largest three test sets with n=256, 512 and 1024 proteomes is almost O(n^2) instead of O(n). I suspect the data points have too large errors. This is presumably also the reason why with

n=1024, FastOMA took longer than the cutoff of 168 hours while OF3 finished before, given that they two tools ran at the same speed for n=256 and n=512 and FastOMA was even slightly faster than OF3 for n=128 and n=64. Therefore, the benchmark should be run with several repeats to reduce error.

Also, it would be important to see the scaling for larger datasets of 2048 and 4096 proteomes, given the need to analyse huge sets from the Earth BioGenome and Darwin Tree of Life projects. In FastOMA. This is particularly important to see how memory scales beyong 1024, given that OF3 appears to have a worse than O(n^2) dependence, while FastOMA has almost constant memory. What is this scaling due to? It looks from this graph as if FastOMA could easily scale to 4096 species and beyond while OF3 will be strongly limited by its O(n^2) memory requirements (Fig. 4b).

2. Given that OF3 and FastOMA are currently the only tools able to run on large datasets, a thoughtful discussion with direct comparison of the strengths and weaknesses of both tools, including the output information provided to the user, would be useful. For example, OMA gives back a hierarchy of clusters of orthologous proteins, with each hierarchy level corresponding to an ancestral node in the species tree, while OF3 gives just a single level. Also, while OMA uses a precomputed database of ortholog groups and requires species trees as input, OF3's approach of computing everything on the fly certainly has some advantage (and disadvantages) over FastOMA's approach?

3. A major modification relative to OF2 is the way how thresholds for the normalized scores between proteins are calculated that define whether an edge is drawn between the protein nodes or not. The explanation of this procedure (lines 286 – 300) is very obscure. For example, line 289: "every RBH observed can be used to estimate a cut-off for the most distant gene in that orthogroup." How exactly is this done? Line 293: "these will each provide multiple independent estimates of the extent of the orthogroup." How do these independent estimates give rise to a single threshold? Do you take the arithmetic average? Or the minimum? Line 294: "a pair of sequences is connected within the graph if the NBS between those sequences fall within that gene-specific and species-specific cut-off." Again, how are the gene-specific and species-specific cut-offs combined? What is NBS?

Minor points:

4. Most modern CPUs have more than 16 cores. It would be useful to see how OF3 and FastOMA runtime scales for 8, 16, 32, 62 cores.

5. The same colors should be used for the same tools in Figures 4 and 5. Also, a color-blind person will not be able to read these graphs. Please use different plotting symbols in addition, and make them big enough to be able to distinguish the colors and shapes clearly.

6. How were the MCL algorithm parameters trained?

7. The QfO benchmark datasets VGNC, SwissTree and TreeFam must have used some orthology inference tools themselves, haven't they. Is there not some bias in favor of methods used in their construction?

8. Lines 145: Mention that the core set is selected by the SHOOT method to be maximally representative. Line 161: AMD Processers => AMD processors or AMD CPUs. Line 161, Fig 4b gb => GB or GBi

Reviewer #2 (Remarks to the Author):

In this manuscript, Emms et al. describe an updated version of the popular software tool OrthoFinder. Authors demonstrate that OrthoFinder v3 outperforms existing tools (including previous versions of OrthoFinder) in both scalability and accuracy. Importantly, a new implementation allows near linear orthogroup delineation by mapping new genomes to a reference orthogroup set reconstructed from a subset of the data. In a time when tens or hundreds of new genomes are released every week, OrthoFinder seems to be an outstanding resource for the evolutionary biology/comparative genomics community. Overall, I really enjoyed reading the manuscript, which is very well written, with rigorous benchmarks, and clear illustrative figures to exemplify new algorithms. Of note, I particularly liked that developers now made a website with clear step-by-step tutorials. Nevertheless, I noticed a few unclear things in the paper, and I believe that addressing them would make the paper clearer to readers and the software more stable.

(1) Authors show that OrthoFinder3_linear is much more scalable than all other tools while preserving (to some extent) accuracy. Specifically, authors show that OrthoFinder3_linear underperformed OrthoFinder3_align, but I wonder if users should worry about to what extent the gain in speed impacts accuracy. Could authors give clear guidelines in the paper? For several hundreds of genomes, using the 'linear' mode seems like an obvious choice; for ~100 genomes, however, the 'linear' mode would be much faster, but with a slightly poorer accuracy (based on benchmarks). In this case, is it generally recommended to go with the 'linear' mode, or should one use the 'align' mode whenever possible? Where should users draw the line?

(2) Along the same lines of my previous point, authors say that "OrthoFinder v3 Linear only slightly underperformed compared to the non-scalable version", but are there variations per gene family? It could be that total differences are small, but some gene families (e.g., rapidly diverging) are particularly more sensitive to the choice of mode. Could authors try to somehow break this comparison in a more fine-grained view?

(3) This could be a consequence of the software development landscape in Python (no central repository that peer-reviews code), but I find it really surprising that a widely used tool like OrthoFinder does not have unit tests. Unit tests are one of the most fundamental best practices in software development, as they ensure functions return what they should return. This is especially important for OrthoFinder v3, since several new implementations have been added, so users have no guarantee that things will work as expected. Many academic users of OrthoFinder probably rely on a centrally installed version in an HPC. Without unit tests, it's very likely that bugs will come out and HPC administrators would have to install upgrades with bug fixes several times. Could authors (and lead developers) take the time to add at least 80% of unit test coverage? This would ensure that the community can (at least more) safely rely on OrthoFinder.

(4) Also on software development: a best practice when writing software documentation is to use a small example data set, so that code chunks can be actually executed. This is not the case for OrthoFinder v3. While the documentation is mostly clear, code chunks are not executed, and output files are described with screenshots, which is problematic for several reasons. There's no guarantee that such screenshots faithfully represent what the code will produce, especially in the long run (after bug fixes and new implementations). Actually executing the code can also be helpful to show users what a 'normal' OrthoFinder run (without errors) should look like, and how to navigate the directory tree with output files. These days, executing code in documentation can be easily done with GitHub Actions, so that the website (and docs) would be re-created after every push and PR merge.

Version 1:

Decision Letter:

Our ref: NMETH-A61931A

10th Feb 2026

Dear Dr. Kelly,

Thank you for submitting your revised manuscript "OrthoFinder: scalable phylogenetic orthology inference for comparative genomics" (NMETH-A61931A). It has now been seen by the original referees and their comments are below. The reviewers find that the paper has improved in revision, and therefore we'll be happy in principle to publish it in Nature Methods, pending minor revisions to comply with our editorial and formatting guidelines.

We are now performing detailed checks on your paper and will send you a checklist detailing our editorial and formatting requirements within two weeks or so. Please do not upload the final materials and make any revisions until you receive this additional information from us.

TRANSPARENT PEER REVIEW
Nature Methods offers a transparent peer review option for new original research manuscripts submitted from 17th February 2021. We encourage increased transparency in peer review by publishing the reviewer comments, author rebuttal letters and editorial decision letters if the authors agree. Such peer review material is made available as a supplementary peer review file. **Please state in the cover letter 'I wish to participate in transparent peer review' if you want to opt in, or 'I do not wish to participate in transparent peer review' if you don't.** Failure to state your preference will result in delays in accepting your manuscript for publication.

Please note: we allow redactions to authors' rebuttal and reviewer comments in the interest of confidentiality. If you are concerned about the release of confidential data, please let us know specifically what information you would like to have removed. Please note that we cannot incorporate redactions for any other reasons. Reviewer names will be published in the peer review files if the reviewer signed the comments to authors, or if reviewers explicitly agree to release their name. For more information, please refer to our FAQ page.

ORCID
IMPORTANT: Non-corresponding authors do not have to link their ORCIDs but are encouraged to do so. Please note that it will not be possible to add/modify ORCIDs at proof. Thus, please let your co-authors know that if they wish to have their ORCID added to the paper they must follow the procedure described in the following link prior to acceptance:
https://www.springernature.com/gp/researchers/orcid/orcid-for-nature-research

Author names using non-Roman characters
Nature Portfolio journals can support presentation of author names using non-Roman characters in the HTML version of the article. If you wish to, please include author names in parentheses after the Roman-character spelling; see example online here. Currently supported scripts are: Arabic, Chinese, Cyrillic, Devanagari, Greek, Hebrew, Hangul, Japanese and Persian. You will be asked to verify the rendering is correct at proof stage.

Thank you again for your interest in Nature Methods. Please do not hesitate to contact me if you have any questions. We will be in touch again soon.

Sincerely,

Lin Tang, PhD
Senior Editor
Nature Methods

Reviewer #1 (Remarks on code availability):

All issues have been addressed satisfactorily.

Reviewer #2 (Remarks to the Author):

I was reviewer #2 in the first review, and I'm glad to see that authors have satisfactorily addressed all points I raised. Hence, I believe the manuscript is ready for publication.

Reviewer #2 (Remarks on code availability):

The code is well written, and authors have implemented changes (unit tests, changes to docs, a minimal reproducible example, GH Actions workflow) based on my previous review.

Version 2:

Decision Letter:

19th May 2026

Dear Professor Kelly,

We apologize for the delay in responding since our team was previously short-staffed. Thank you very much for your patience when we check the revised version of your Article, "OrthoFinder: Improved phylogenetic orthology inference with enhanced accuracy and scalability". I am pleased to inform you that this paper has now been accepted for publication in Nature Methods. The received and accepted dates will be 15th Jul 2025 and 19th May 2026. This note is intended to let you know what to expect from us over the next month or so, and to let you know where to address any further questions.

Over the next few weeks, your paper will be copyedited to ensure that it conforms to Nature Methods style. Once your paper is typeset, you will receive an email with a link to choose the appropriate publishing options for your paper and our Author Services team will be in touch regarding any additional information that may be required. It is extremely important that you let us know now whether you will be difficult to contact over the next month. If this is the case, we ask that you send us the contact information (email, phone and fax) of someone who will be able to check the proofs and deal with any last-minute problems.

After the grant of rights is completed, you will receive a link to your electronic proof via email with a request to make any corrections within 48 hours. If, when you receive your proof, you cannot meet this deadline, please inform us at rjsproduction@springernature.com immediately.

Authors may need to take specific actions to achieve compliance with funder and institutional open access mandates. If your research is supported by a funder that requires immediate open access (e.g. according to Plan S principles or the NIH public access policy) then you should select the gold OA route, and we will direct you to the compliant route where possible. Because authors warrant under our subscription licensing terms that they haven’t committed to licensing any version of their article under a licence inconsistent with the terms of our agreement – including the applicable embargo period – publication under the subscription model isn’t suitable for authors whose funders require no embargo.

If you have any questions about our publishing options, costs, Open Access requirements, or our legal forms, please contact ASJournals@springernature.com

If you have posted a preprint on any preprint server, please ensure that the preprint details are updated with a publication reference, including the DOI and a URL to the published version of the article on the journal website.

You may wish to make your media relations office aware of your accepted publication, in case they consider it appropriate to organize some internal or external publicity. Once your paper has been scheduled you will receive an email confirming the publication details. This is normally 3-4 working days in advance of publication. If you need additional notice of the date and time of publication, please let the production team know when you receive the proof of your article to ensure there is sufficient time to coordinate. Further information on our embargo policies can be found here: https://www.nature.com/authors/policies/embargo.html

To assist our authors in disseminating their research to the broader community, our SharedIt initiative provides you with a

unique shareable link that will allow anyone (with or without a subscription) to read the published article. Recipients of the link with a subscription will also be able to download and print the PDF.

As soon as your article is published, you will receive an automated email with your shareable link.

If you are active on Twitter/X or Bluesky, please e-mail me your and your coauthors' handles so that we may tag you when the paper is published.

You can now use a single sign-on for all your accounts, view the status of all your manuscript submissions and reviews, access usage statistics for your published articles and download a record of your refereeing activity for the Nature journals.

Please note that you and any of your coauthors will be able to order reprints and single copies of the issue containing your article through Nature Portfolio's reprint website, which is located at http://www.nature.com/reprints/author-reprints.html. If there are any questions about reprints please send an email to author-reprints@nature.com and someone will assist you.

Please feel free to contact me if you have questions about any of these points. Thank you very much for publishing your paper at Nature Methods!

Best regards,

Lin Tang, PhD
Senior Editor
Nature Methods

** Visit the Springer Nature Editorial and Publishing website at www.springernature.com/editorial-and-publishing-jobs for more information about our career opportunities. If you have any questions please click here.**

Open Access This Peer Review File is licensed under a Creative Commons Attribution 4.0 International License, which permits use, sharing, adaptation, distribution and reproduction in any medium or format, as long as you give appropriate credit to the original author(s) and the source, provide a link to the Creative Commons license, and indicate if changes were made. In cases where reviewers are anonymous, credit should be given to 'Anonymous Referee' and the source.
The images or other third party material in this Peer Review File are included in the article's Creative Commons license, unless indicated otherwise in a credit line to the material. If material is not included in the article's Creative Commons license and your intended use is not permitted by statutory regulation or exceeds the permitted use, you will need to obtain permission directly from the copyright holder.
To view a copy of this license, visit https://creativecommons.org/licenses/by/4.0/

OrthoFinder response to Reviewers

We thank the editor and both reviewers for their insightful and constructive comments. In the revised manuscript we have addressed each of the comments that were made. The changes that were made are described in the point-by-point responses below. The changes have strengthened the manuscript.

In this document, we use **black text** for the reviewers' comments, and **blue text** for our response.

Reviewer #1 (Remarks to the Author):

The manuscript describes the OrthoFinder3 method for orthology inference. It builds on the highly successful OrthoFinder2 software by the same lab, which has garnered over 6000 citations since 2019. Input is a set of proteomes, each comprising the protein sequences encoded in the genome for a certain species. As in version 2, the output is a list of orthogroups, ortholog pairs between species, species trees etc. The main improvement over version 2 is the speed improvement (roughly a factor 13 for n = 256 proteomes) and a slightly better scaling with the n. This was achieved using a similar idea as in FastOMA (Nature Methods 2025): to run the slow O(n^2) all-vs-all similarity search needed for a first clustering of input proteins into tentative orthogroups only on a precomputed species set (FastOMA) or on small subset of the input proteomes (Orthofinder3). The proteins from the other species/proteomes are then assigned to one of the tentative orthogroups. In the next step, both methods run their magic to split the resulting tentative orthogroups up into the actual, orthogroups returned to the user.

Orthofinder3 has nearly the same performance as Orthfinder2 while being much faster. It has very similar speed as FastOMA, and these two tools are the only ones able to run on large input sets (>1000 species) in acceptable time (Fig. 4). However, OF3's accuracy according to various measures in the standardized Quest for Orthologs (QfO) benchmark is quite a bit better than that of FastOMA (Fig 5).

The methodological improvements are rather simple and reasonable and rely partly on integrating previous work such as SHOOT into OF3, but the end result, given the enormous popularity of OF2 due to its unmatched accuracy and usablility, could make OF3 with its improved speed very popular as well, if the scaling issues for memory and a few other points detailed below are addressed properly.

We thank the reviewer for their positive and supportive review of our work. We have addressed the concerns regarding the scalability of the method in the point-by-point responses below.

Major issues

1. The better speed and possibly better scaling (theoretically down to n instead of n^2) is the main selling point of OrthoFinder. However, while in the speed benchmark of FastOMA (Nature Methods 2025, Fig. 2c) one could see a clean O(n^2) scaling of OF2 and other tools and O(n) for FastOMA, the speed benchmark in the present study does not show any clean scaling at all. In fact, unexpectedly the scaling of

FastOMA and OF3 for the largest three test sets with n=256, 512 and 1024 proteomes is almost O(n^2) instead of O(n).

We thank the reviewer for raising this key issue. We were also surprised by this result with FastOMA and it took some time to figure out why it was happening. The reviewer is correct that the figure in the original FastOMA paper suggests a near linear run time. However, this performance characteristic is an artefact of how the example data was chosen and is not representative of the method performance in real world applications. Specifically, the near linear run-time was achieved by the use of a non-resolved species tree. FastOMA makes use of phylogenetic tree for its calculations and the number of calculations performed scales with the number of branches in the phylogenetic tree. To achieve the near linear run-time the authors did not include all the branches in their phylogenetic tree. Instead they used only a partial set, for example in the largest dataset the phylogenetic tree should have had 2178 internal branches but the tree they used only had 1107 internal branches. As the method infers hierarchical orthogroups (i.e. an orthogroup for every bipartition in the input tree) the lack of resolution in the species tree enables the method to skip calculations it would otherwise need to make to infer orthologs and orthogroup and thus enables it to run faster. When FastOMA is run using a resolved species tree (as is the way a user would expect to use it, and as is the way that it should be used) the method displays non-linear runtime as shown in our manuscript. It is very important to note here that when FastOMA is run with a non-resolved species tree the accuracy of the FastOMA method is severely reduced (as discussed by the authors in their original FastOMA manuscript) with the vast majority of ortholog inferences either missing or incorrect. Accordingly, for accuracy benchmarking in the FastOMA paper the authors ran FastOMA with a fully resolved species tree which improves the accuracy performance (although it is still substantially worse than competitor methods). The authors did not report the run-times for FastOMA using a resolved species tree in their paper if they did it would have been non-linear.

I suspect the data points have too large errors. This is presumably also the reason why with n=1024, FastOMA took longer than the cutoff of 168 hours while OF3 finished before, given that they two tools ran at the same speed for n=256 and n=512 and FastOMA was even slightly faster than OF3 for n=128 and n=64. Therefore, the benchmark should be run with several repeats to reduce error.

We thank the reviewer for raising this point. We now provide the error bars for both FastOMA and OrthoFinder in the Supplementary File 4 Figure 2. The error bars are very small as the run time is highly reproducible between repeats of the same analysis.

The reviewer noted that FastOMA is faster than OF3 for the 64 and 128 species datasets. While we fully agree with this (although noting OrthoFinder is faster than FastOMA for all other dataset sizes including 4, 8, 16, 32, 256, 512, and 1024 species), the runtime data that is presented for up to 64 species does not use our new scalable method. The scalable method is only used for species sets larger than 64. This is explained in lines 169-172 of the manuscript. We agree with the reviewer

that for smaller numbers of species the runtimes are similar. However, the runtimes diverge and the speed benefit of OrthoFinder increases with increasing species number. For example, FastOMA took 198 hours to finish the 1,024 species run compared to 133 hours for OrthoFinder. This information has been provided in the text of the revised manuscript. We have kept the 7 day cut off for the figures in the manuscript because “what can I achieve in a week” gives a very intuitive timeframe to help users understand the performance characteristics of the different methods. We would also like to stress that even if the run times were similar on some datapoints, OrthoFinder is substantially more accurate than FastOMA and both the run time and accuracy should be considered here.

Also, it would be important to see the scaling for larger datasets of 2048 and 4096 proteomes, given the need to analyse huge sets from the Earth BioGenome and Darwin Tree of Life projects. In FastOMA. This is particularly important to see how memory scales beyong 1024, given that OF3 appears to have a worse than O(n^2) dependence, while FastOMA has almost constant memory. What is this scaling due to? It looks from this graph as if FastOMA could easily scale to 4096 species and beyond while OF3 will be strongly limited by its O(n^2) memory requirements (Fig. 4b).

We completely agree that scaling to several thousand genomes is important for the future of orthology inference – particularly given the groundbreaking work by projects such as Earth BioGenome and Darwin Tree of life. However, whilst this data is coming soon, we currently lack enough high-quality eukaryotic genomes to reach 4096 species in this paper (Ensembl has 1,789, for example). To address the reviewers important point regarding scalability and the performance of OrthoFinder and FastOMA with increased data sizes, we have conducted an analysis on a larger numbers of bacterial genomes for which larger numbers are publicly available. We compiled a dataset of 2048 and 4096 bacteria proteomes subject them to OrthoFinder scalable algorithm in exactly the same manner as described in the original manuscript for consistency. For comparison we also tested FastOMA on these datasets also using the same number of compute cores. OrthoFinder was able to successfully complete the 4096 proteome dataset in 13 days and 15 hours requiring approximately 500GB of RAM, and completed the 2048 dataset in only 50 hours also requiring 500GB of RAM. The significantly longer run time for 4096 species was due to the workflow reaching the full amount of available RAM on our servers (500GB) and making use of the swap space for additional memory requirements. FastOMA took 15 days to run the 2048 species analysis and was not able to complete the 4096 bacteria during the time we had available to respond to these reviewers comments.

2. Given that OF3 and FastOMA are currently the only tools able to run on large datasets, a thoughtful discussion with direct comparison of the strengths and weaknesses of both tools, including the output information provided to the user, would be useful. For example, OMA gives back a hierarchy of clusters of orthologous proteins, with each hierarchy level corresponding to an ancestral node in the species tree, while OF3 gives just a single level. Also, while OMA uses a precomputed database of ortholog groups and requires species trees as input, OF3’s approach of

computing everything on the fly certainly has some advantage (and disadvantages) over FastOMA's approach?

We agree that such a comparison would be useful. We have added a new section discussing of the strengths and weakness of OrthoFinder vs. FastOMA is on lines 257-268 of the revised manuscript.

3. A major modification relative to OF2 is the way how thresholds for the normalized scores between proteins are calculated that define whether an edge is drawn between the protein nodes or not. The explanation of this procedure (lines 286 – 300) is very obscure. For example, line 289: "every RBH observed can be used to estimate a cut-off for the most distant gene in that orthogroup." How exactly is this done? Line 293: "these will each provide multiple independent estimates of the extent of the orthogroup." How do these independent estimates give rise to a single threshold? Do you take the arithmetic average? Or the minimum? Line 294: "a pair of sequences is connected within the graph if the NBS between those sequences fall within that gene-specific and species-specific cut-off." Again, how are the gene-specific and species-specific cut-offs combined? What is NBS?

We thank the reviewer for pointing out this oversight. We have revised the section of the manuscript that explains this process (lines 317-357).

BLAST scores are influenced by sequence length, with longer sequences tending to produce higher scores. For OF1 and OF2, we used a novel method to normalize BLAST scores to account for this bias. Specifically, this method creates several bins of different sequence length, and fits a linear model to the top scoring 5% of hits in each bin. This allows us to transform raw BLAST scores to account for the sequence length bias. This transformation also accounted for phylogenetic distance – with best hits between distant species getting on average the same score as best hits between closely-related species.

With the new OF3 method, we first perform a 'core' run, and then 'assign' new genes to the core orthogroups. We therefore don't have the sample size to fit reliable linear models to inform the normalization. Instead, we introduce a new method to normalize bit-scores. Importantly, we now separate the solution to the length bias and phylogenetic distance problems into two distinct parts. First, we deal with sequence length by normalising bit-scores between two sequences using the geometric mean of the length of the query and hit sequences. These length-normalised bit-scores are termed NBS. Two genes x and y can be reciprocal best hits (RBS) to each other based on their NBS.

We then deal with the phylogenetic distance problem. The solution has two parts – one of which is species-specific, and one of which is gene-specific. For the species-specific part, let us consider a triplet of species (X,Y,Z) with a set of genes (e.g x,y,z). For all genes in X that have RBH in both Y & Z, we calculate the ratio $\mathbf{R}_{XYZ}$ of NBS between these pairs (x->y / x->z). To obtain a single value for a species triplet, we define $\mathbf{R}^*_{XYZ}$ as the 90th percentile of this set of ratios. To obtain a value for a single species pair X and Z, we use the minimum value across all Y species to define $\mathbf{R}^*_{XY}$. This is deliberately chosen to be an inclusive value, as our phylogenetic delineation

method will break up erroneously-fused orthogroups at a later stage in the algorithm. This value is species-specific, and can be applied across all orthogroups.

For the gene-specific part, let us now consider a specific RBH between gene x and gene z, with an NBS of B_{xz}. We use this gene-specific score measure to weight our species-specific measure R^*_{XZ} and determine the extent of the orthogroup. Specifically, the most distant ortholog in species Y must have a score greater than $B_{xz}R^*_{XZ}$. This threshold combines the species-specific and gene-specific cut-offs into a single measure. When we have multiple independent estimates of the extent of an orthogroup (e,g, if a gene has RBH in multiple species), we use an inclusive approach where a pair of genes is connected if it passes any of the thresholds.

Minor points:

4. Most modern CPUs have more than 16 cores. It would be useful to see how OF3 and FastOMA runtime scales for 8, 16, 32, 62 cores.

We thank the reviewer for this suggestion. In response, we have now preformed an additional scalability analysis where we have run OrthoFinder and FastOMA on a range of threads (4, 8, 16, 32, 64, 128) on 64 proteomes. We chose 64 proteomes as this is the dataset size in our analysis where the two methods perform most similarly, and thus does not bias the results towards our method (which performs better at almost all other dataset sizes). For this test we split the input dataset into a core of 16 species and then an additional set of 48 proteomes. The time shown for OrthoFinder therefore shows the time taken to build the 16-proteome core set **plus** the time taken to assign 48 proteomes to this core set. (It should be noted that the time taken to build the FastOMA dataset is not included in the FastOMA run time). Both tools have diminishing returns for run time when adding extra multiprocessing resources, with an initial benefit in runtime for increasing threads followed by a plateau after 32 threads. Memory usage for both tools increased approximately linearly with the number of threads. The scaling behaviour of OF3 and FastOMA is comparable for runtime and memory. We have added these additional Figures in new Supplemental File 4 Figure 1 and added additional comments addressing this to the revised manuscript (lines 187-192).

5. The same colors should be used for the same tools in Figures 4 and 5. Also, a color-blind person will not be able to read these graphs. Please use different plotting symbols in addition, and make them big enough to be able to distinguish the colors and shapes clearly.

We thank the reviewer for this suggestion. We have changed these figures – and now use color-blind friendly palettes, consistent colours, and different plot symbols.

6. How were the MCL algorithm parameters trained?

The only parameter in MCL is the inflation factor. This was trained in the OF1 paper (Emms & Kelly, 2015), where we showed that accuracy of OrthoFinder is robust to changes in inflation factor. OrthoFinder 3 uses a value I=1.2, which leads to larger clusters. Our previous training showed that this value leads to low precision whilst maximising recall. Because we have the new phylogenetic delineation method to

split erroneously fused orthogroups, it makes sense to choose such a value. We prioritize recall, and let the delineation algorithm correct the over-clustering of some orthogroups. We have added a sentence explaining this, on lines 360-361.

QfO benchmark datasets VGNC, SwissTree and TreeFam must have used some orthology inference tools themselves, haven't they. Is there not some bias in favor of methods used in their construction?

This is a very interesting point raised by the reviewer. In all cases the reference trees in question are not inferred using any of the orthology methods tested in this paper. These trees were assembled using *ad hoc* methods (largely using BLAST or HMM searches coupled to a variety of different phylogenetic tree inference methods and extensive manual curation). Thus, the diversity of methods used in these benchmarks does not align more closely with any method tested in our paper here and does not bias towards any particular method.

8. Lines 145: Mention that the core set is selected by the SHOOT method to be maximally representative. Line 161: AMD Processers => AMD processors or AMD CPUs. Line 161, Fig 4b gb => GB or GBi

Thank you for spotting this, it is now corrected.

Reviewer #2 (Remarks to the Author):

In this manuscript, Emms et al. describe an updated version of the popular software tool OrthoFinder. Authors demonstrate that OrthoFinder v3 outperforms existing tools (including previous versions of OrthoFinder) in both scalability and accuracy. Importantly, a new implementation allows near linear orthogroup delineation by mapping new genomes to a reference orthogroup set reconstructed from a subset of the data. In a time when tens or hundreds of new genomes are released every week, OrthoFinder seems to be an outstanding resource for the evolutionary biology/comparative genomics community. Overall, I really enjoyed reading the manuscript, which is very well written, with rigorous benchmarks, and clear illustrative figures to exemplify new algorithms. Of note, I particularly liked that developers now made a website with clear step-by-step tutorials. Nevertheless, I noticed a few unclear things in the paper, and I believe that addressing them would make the paper clearer to readers and the software more stable.

Thank you for your positive support! We hope that OrthoFinder will continue to be a useful resource for the community.

(1) Authors show that OrthoFinder3_linear is much more scalable than all other tools while preserving (to some extent) accuracy. Specifically, authors show that OrthoFinder3_linear underperformed OrthoFinder3_align, but I wonder if users should worry about to what extent the gain in speed impacts accuracy. Could authors give clear guidelines in the paper? For several hundreds of genomes, using the 'linear' mode seems like an obvious choice; for ~100 genomes, however, the 'linear' mode would be much faster, but with a slightly poorer accuracy (based on benchmarks). In this case, is it generally recommended to go with the 'linear' mode,

or should one use the ‘align’ mode whenever possible? Where should users draw the line?

We thank the Reviewer for this suggestion. To address this we now include advice on the github page and our website. Specifically, our advice is to use the scalable method when analysing >100 species. This is based on the balance of scalability and accuracy. As OrthoFinder is (and has always been) developed in the public domain and made available prior to publication, we will continue to update the guidance of when to use the scalable vs the original method as we continue to advance the method.

(2) Along the same lines of my previous point, authors say that “OrthoFinder v3 Linear only slightly underperformed compared to the non-scalable version”, but are there variations per gene family? It could be that total differences are small, but some gene families (e.g., rapidly diverging) are particularly more sensitive to the choice of mode. Could authors try to somehow break this comparison in a more fine-grained view?

We fully agree with the reviewer here. However, the community currently lacks the benchmark datasets to be able to say what feature of a particular orthogroup (its size, gene length, species distribution, evolutionary rate, duplication rate, indel rate, etc) causes errors in orthology inference. We attempted to do this with the OrthoBench dataset but with only 70 reference orthogroups there was not sufficient data to be able to reach any conclusions about which feature of an orthogroup make it more or less challenging. We think that the reviewer is correct, and that it is an important challenge for the orthology inference community to gather together and develop such a database - like we did for the quest for orthologs ortholog benchmarking. However, we believe that this is best as a community activity to ensure that it is not biased toward any particular orthology inference method and thus its construction and evaluation fall outside of the work presented here.

(3) This could be a consequence of the software development landscape in Python (no central repository that peer-reviews code), but I find it really surprising that a widely used tool like OrthoFinder does not have unit tests. Unit tests are one of the most fundamental best practices in software development, as they ensure functions return what they should return. This is especially important for OrthoFinder v3, since several new implementations have been added, so users have no guarantee that things will work as expected. Many academic users of OrthoFinder probably rely on a centrally installed version in an HPC. Without unit tests, it’s very likely that bugs will come out and HPC administrators would have to install upgrades with bug fixes several times. Could authors (and lead developers) take the time to add at least 80% of unit test coverage? This would ensure that the community can (at least more) safely rely on OrthoFinder.

We thank the reviewer for this helpful comment. In response to this we have implemented a broad suite of integration tests that will be invaluable to our large community of users, as well as assisting us as we develop OrthoFinder into the future. At present, these integration tests cover approximately 70% of the

OrthoFinder code base, including all newly introduced functionality in OrthoFinder v3. Our integration tests assess both the correction function of the full OrthoFinder pipeline under a variety of command-line configurations and the behaviour of targeted sets of code units. We have created expected outcomes by designing a curated ground truth dataset with an expected outcome against which OrthoFinder runs can be compared. Further details regarding the approach to the test functions can be found in Supplementary File 5.

We agree that further comprehensive package of unit testing of individual functions will further strengthen code reliability. As part of the upcoming major code refactor for the next version of OrthoFinder, we are actively introducing unit testing as a core part of our software development ethos. We thank the reviewer for making this suggestion which, although it does not influence any of the results presented in the manuscript, does make OrthoFinder much more resilient, robust, and will ultimately make it easier for us to develop the code in the future.

(4) Also on software development: a best practice when writing software documentation is to use a small example data set, so that code chunks can be actually executed. This is not the case for OrthoFinder v3. While the documentation is mostly clear, code chunks are not executed, and output files are described with screenshots, which is problematic for several reasons. There's no guarantee that such screenshots faithfully represent what the code will produce, especially in the long run (after bug fixes and new implementations). Actually executing the code can also be helpful to show users what a 'normal' OrthoFinder run (without errors) should look like, and how to navigate the directory tree with output files. These days, executing code in documentation can be easily done with GitHub Actions, so that the website (and docs) would be re-created after every push and PR merge.

Thank you for this helpful suggestion. We agree that having executable examples and automatically tested documentation is a valuable practice. We already use GitHub Actions to run OrthoFinder with every push and pull request merge. We also provide a minimal example dataset to provide consistent results for users who wish to test OrthoFinder. To address directly the reviewer's concerns, we have added additional GitHub Actions to our tutorial's documentation. These will automatically output data and file listings for an OrthoFinder run on our example dataset, ensuring that any updates to OrthoFinder are automatically reflected in the tutorials. This enables users to view a reproducible listing of expected outcomes and ensures that all documentation corresponds accurately to both current and future OrthoFinder outputs. We have added these actions for the complete beginner tutorial section ensuring that new users are presented with reproducible example structures and data. The tutorials on our website were originally designed for a beginner audience to guide users through the downloading input data to running OrthoFinder and interpreting results for larger, "real" datasets. The changes that we have made here will benefit the user community and provide consistency for future OrthoFinder updates. We thank the reviewer for motivating us to make these changes to the website.
